# YY1-induced USP43 drives ferroptosis suppression by FASN stabilization and subsequent activation of SLC7A11 in ovarian cancer

**DOI:** 10.1038/s41419-025-07886-5

**Published:** 2025-09-01

**Authors:** Tianyi Zhao, Xiaojun Chen, Jiangchun Wu, Siyu Chen, Yu Gan, Chaohua Liu, Xinyu Ha, Yangjun Wu, Xiang Zhou, Yong Wu, Xiaohua Wu

**Affiliations:** 1 Department of Gynecologic Oncology, Fudan University Shanghai Cancer Center, Shanghai, China; 2 Department of Obstetrics and Gynecology, Qilu Hospital, Cheeloo College of Medicine, Shandong University, Jinan, Shandong China; 3 Department of Oncology, Shanghai Medical College, Fudan University, Shanghai, China; 4 Fudan University Shanghai Cancer Center and Institutes of Biomedical Sciences, Fudan University, Shanghai, China; 5 Key Laboratory of Breast Cancer in Shanghai, Fudan University Shanghai Cancer Center, Shanghai, China; 6 Shanghai Key Laboratory of Medical Epigenetics, International Co-laboratory of Medical Epigenetics and Metabolism (Ministry of Science and Technology), Institutes of Biomedical Sciences, Fudan University, Shanghai, China

**Keywords:** Ovarian cancer, Cancer metabolism

## Abstract

The ubiquitin-specific protease (USP) family is a major member of the deubiquitinating enzyme family that plays important and diverse roles in multiple tumors. The roles and mechanisms of action of USP family members in ovarian cancer are not well understood. This study aimed to screen all the USP family members and explored the specific function of USP43 in ovarian cancer. The expression levels of USP family members in ovarian cancer were screened using bioinformatics analysis, and the specific function of USP43 was explored through in vitro and in vivo experiments. Functional assays, including cell viability, ferroptosis, and tumor xenograft models, were employed. In short, USP43 drives the ferroptosis suppression by activating the expression of SLC7A11 through FASN-HIF1α pathway. USP43 is an important prognostic factor for ovarian cancer, with its overexpression promoting ovarian cancer progression and its knockdown inhibiting it. Mechanistically, USP43, which is transcriptionally activated by YY1, stabilizes FASN through deubiquitination, and FASN activates SLC7A11 expression by stabilizing HIF1α. Furthermore, the combination of cisplatin and the SLC7A11 inhibitor HG106 significantly inhibits the growth of ovarian tumors. Thus, targeting the USP43-FASN-HIF1α-SLC7A11 axis can inhibit ferroptosis and promote platinum sensitivity in ovarian cancer.

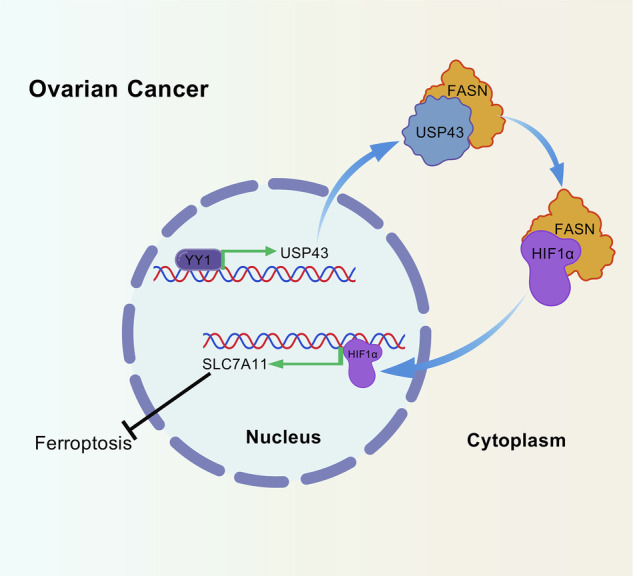

## Introduction

Mortality due to ovarian cancer (OC) ranks fifth among female human tumor-related deaths worldwide, placing a great burden on the economic and healthcare systems, with a median overall survival (OS) of 40.7 months [1]. Platinum–paclitaxel doublet cytotoxic agents have been the first-line standard of care (SOC) for patients with OC for a long time [1]. Although many patients show a transient response to initial platinum-based agents, the development of resistance to platinum becomes inevitable in the vast majority of patients during the disease course [2]. Despite recent therapeutic advances in poly ADP ribose polymerase (PARP) inhibitors and novel promising strategies, the long-term survival of patients with OC remains poor, especially in platinum-resistant cases [3]. Immunotherapy has made remarkable progress in cancer treatment; however, its role in OC treatment is limited [4]. In recent years, the regulation of cell metabolism and cell death has been considered a potential option for tumor treatment [5]. Thus, the discovery of additional therapeutic biomarkers and efficient treatments related to cell metabolism and death is important to meet the unmet treatment needs of OC.

Multiple ubiquitin-specific proteases (USPs) have been identified as promising biomarkers and therapeutic targets in OC. USPs are the most numerous and widely studied family of deubiquitinating enzymes (DUBs), with over 50 members accounting for half of all DUBs [6]. USPs affect a variety of biological processes, including tumor immunity, metabolism, and drug resistance [6, 7], and USP family members are notably involved in cancer initiation and development by protein ubiquitination [8]. USPs act as oncogenes or tumor suppressors owing to their complex functions. For example, USP5 overrides p53-dependent senescence and drives KRAS-induced tumorigenicity by stabilizing nuclear Beclin 1 [9]. USP1 binds to replication forks and promotes the survival of BRCA1-deficient cells [10]. USP7 is a well-characterized member of the USP family and is considered an emerging drug target in cancer [11, 12]. USP7 stabilizes MDM2/MDMX and subsequently mediates p53 degradation [13] and also promotes APC-mutant intestinal hyperproliferation and tumor development [14]. Furthermore, USP7 is a prognostic factor in OC, and one of its inhibitors, P5091, can effectively suppress OC cells [15, 16]. Similarly, USP15 mutation can inhibit the interaction of HP1γ and BARD1, reducing BRCA1/BARD1 retention at DNA double-strand breaks. Thus, USP15 mutation is a candidate biomarker for PARPi in patients with OC but without BRCA1/2 mutations [17].

USP43 is a rarely reported member of the USP family and is associated with tumor proliferation and metastasis [18]. USP43 has been explored as a suppressor of carcinogenesis in breast cancer and as an oncogene that promotes tumor progression in colorectal cancer and bladder cancer [18–20]. In breast cancer, nuclear-localized USP43 binds to the NuRD complex and exerts tumor-suppressive effects by deubiquitinating H2BK120 and suppressing the transcription of EGFR, whereas cytoplasmic retention of USP43 contributes to tumor progression [20]. In contrast, USP43 promotes colorectal cancer cell proliferation, migration, and invasion through its deubiquitination-mediated stabilization of ZEB1 [19]. While in bladder cancer, USP43 was found to have the ability to stabilize c-Myc to promote the glycolysis of cancer cells [18]. Recently, the role of USP43 in regulating OC progression and the underlying mechanisms have been discovered [21], indicating that USP43 might serve as a potential target for the control of OC progression. USP43 could reduce cisplatin sensitivity in OC by stabilizing HDAC2 and activating the wnt/β-catenin signaling pathway [21]. However, the potential effects and molecular mechanisms of USP43 in regulating OC cell metabolism and death remain unknown.

Targeting ferroptosis induction in tumor cells is also a beneficial approach for cancer treatment. Ferroptosis is a type of cell death involving plasma membrane lipid peroxidation and the redox of metal iron ions and differs from other cell death modes [22]. Ferroptosis involves the peroxidation of unsaturated fatty acids on the cell membrane. Lipid metabolic enzymes and plasma membrane transporters participate in ferroptosis regulation [23]. The ratio of saturated to unsaturated fatty acids produced during different fatty acid synthesis processes is the key factor affecting ferroptosis sensitivity. Fatty acid synthase (FASN) utilizes cytosolic acetyl-CoA carboxylase 1 (ACC1), which generates malonyl-CoA to synthesize long-chain saturated fatty acids and reduces the sensitivity to ferroptosis [24]. For example, the activation of oncogenic KRAS amplifies FASN expression, which increases the conversion of long-chain saturated fatty acids in the cell membrane into polyunsaturated fatty acids (PUFAs), leading to ferroptosis resistance [25]. Furthermore, FASN and SCD1 levels were increased in acute myeloid leukemia with FLT3 mutation [26]. Similarly, the ferroptosis inducer erastin promoted ferroptosis by inhibiting the xCT system on the cell surface, which is a cysteine-glutamate transporter that imports cysteine into the plasma. Cystine, an essential substrate for the biosynthesis of glutathione (GSH), is of great importance in inhibiting oxidative stress and ferroptosis [27]. Solute carrier family 7 member 11 (SLC7A11) is the light subunit of the xCT system, whose dysregulation strongly influences sensitivity to ferroptosis [28].

Increasing number of studies have shown that ferroptosis plays an important role in the progression of cancer, especially for drug-resistant tumors, which are more likely to be induced to ferroptosis [29]. However, it is unclear whether USP43 affects ferroptosis and through what mechanism it affects ferroptosis in ovarian cancer cells. Hence, in this study, we aimed to identify the role and potential molecular basis of USP43 in ferroptosis in OC cells through in vitro and in vivo assays and provide new treatment options for ovarian cancer patients.

## Results

### USP43 is specifically overexpressed in ovarian malignant epithelial cells and is associated with poor prognosis in patients with OC

**Fig. 1:**
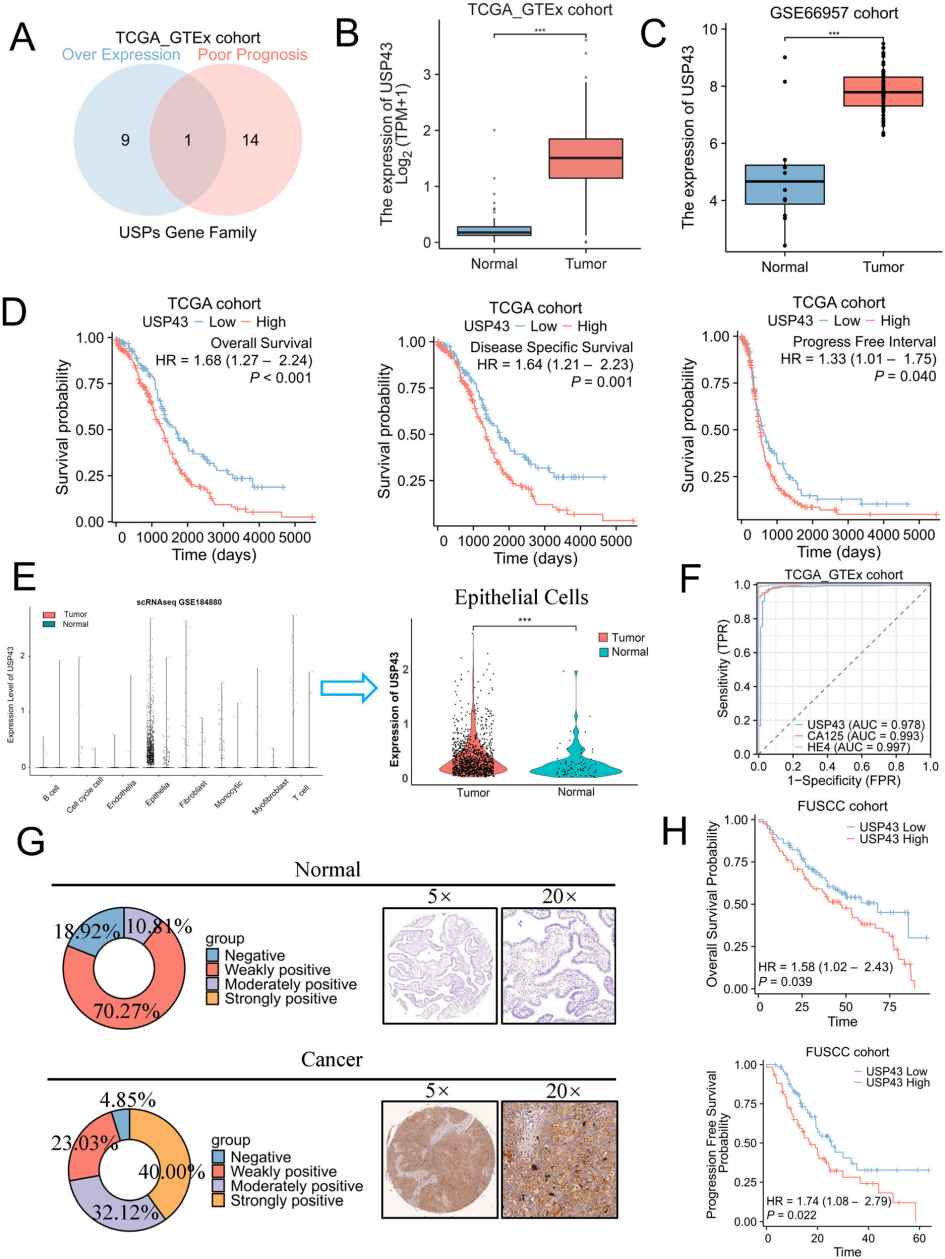
Screening revealed that USP43 is highly expressed in ovarian cancer (OC) and is associated with poor prognosis. **A** Venn diagram shows that only USP43 is overexpressed and positively correlates with poor prognosis in patients with OC. **B** Expression of USP43 in OC and non-matched normal tissues based on the data retrieved from TCGA and GTEx databases. **C** Expression of USP43 in OC and non-matched normal tissues in the GSE66957 cohort. **D** Overall survival, progress-free interval, and disease-specific survival in patients with OC with high and low USP43 expression. **E** USP43 expression in different OC cell types. USP43 expression was higher in malignant epithelial cells than in normal epithelial cells. **F** ROC curves based on USP43 expression for classifying OC versus normal ovarian tissues using TCGA data. **G** Immunohistochemical staining score and representative images of normal and tumor ovarian epithelial tissues stained for USP43. **H** Overall survival and progress-free interval for patients with OC in FUSCC with high and low USP43 expression. Data are presented as mean ± SD.

To discover the role of the USP gene family in OC, we screened all 54 USP genes using The Cancer Genome Atlas (TCGA) and Genotype-Tissue Expression (GTEx) databases. A total of 10 USP family genes were overexpressed in patients with OC, and 15 USP family genes were correlated with poor prognosis in patients with OC. USP43 was the only gene that simultaneously met these two conditions (Fig. 1A). USP43 was barely expressed in normal ovarian tissues but overexpressed in OC tissues (Fig. 1B). We further validated these results using the GSE66957 dataset (Fig. 1C). Patients with high USP43 expression in OC showed significantly shorter overall survival (OS), disease-specific survival (DSS), and progression-free Interval (PFI) than patients with low USP43 expression (Fig. 1D). To further determine the primary cell types expressing USP43, we analyzed single-cell datasets of epithelial OC and normal ovarian tissues. After annotating the cell types, we found that USP43 was specifically expressed in epithelial cells, and its expression in malignant epithelial cells was significantly higher than that in normal ovarian epithelial cells (Fig. 1E and S1A-C). Receiver Operating Characteristic (ROC) curve analysis showed that the sensitivity and specificity of USP43 as a diagnostic marker for OC were comparable to those of MUC16 and WFDC2, which are widely used in clinical practice (Fig. 1F). Immunohistochemical results from the human protein atlas (HPA) database showed that the protein level of USP43 in malignant tumor tissues was also higher than that in normal ovarian tissues (Fig. S1D). We further evaluated the changes in USP43 expression in patients with OC by performing immunohistochemistry (IHC) assays on normal and epithelial OC tissues from the Fudan University Shanghai Cancer Center (FUSCC) clinical cohort. The extent of immunohistochemical staining of USP43 in epithelial OC tissues was significantly higher than that in normal ovarian tissues (Fig. 1G). Further, patients with OC and high USP43 immunohistochemical staining scores in FUSCC had shorter OS and PFS than patients with low staining scores (Fig. 1H).

### Induction or downregulation of USP43 modifies malignant phenotype of OC cells in vitro

**Fig. 2:**
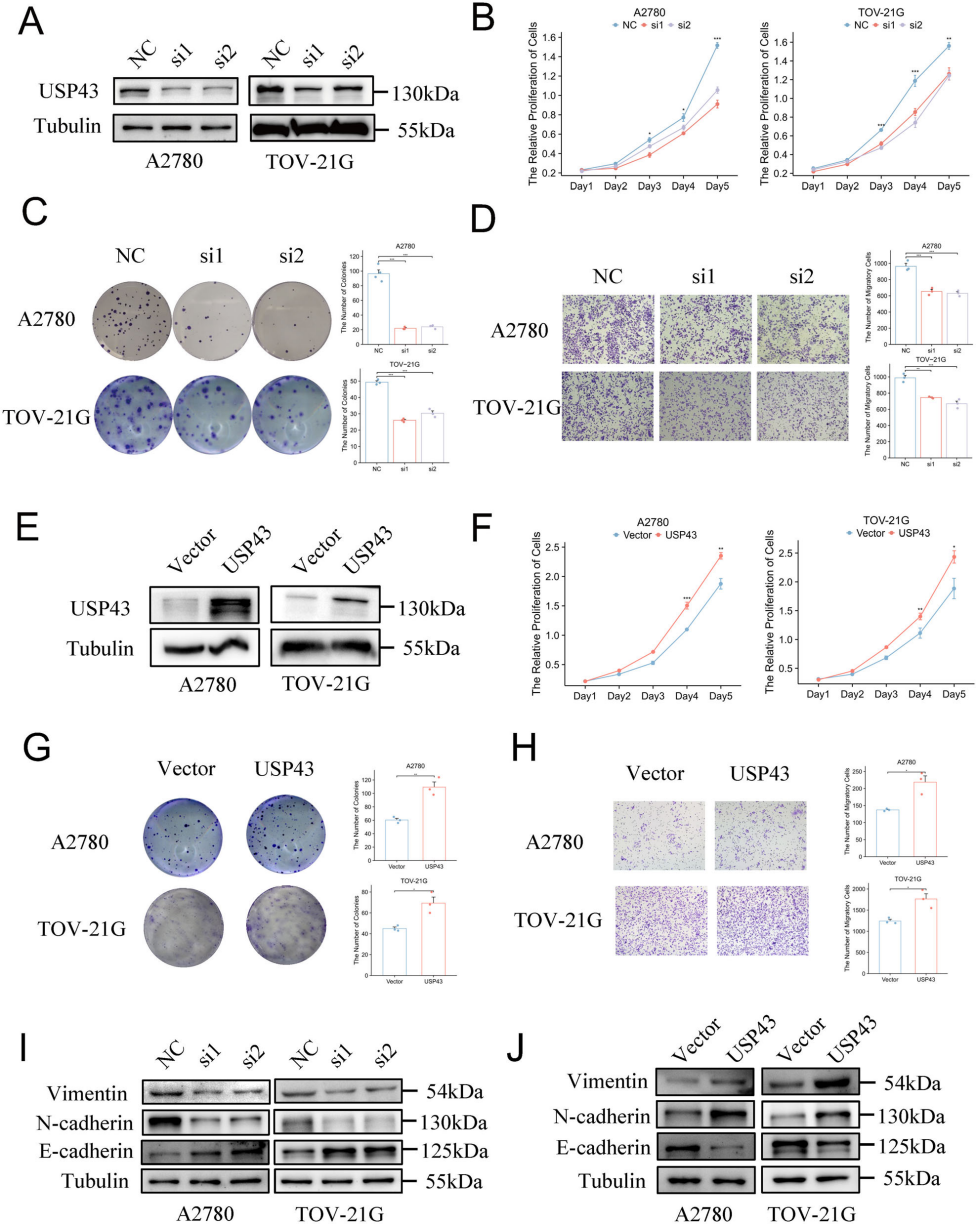
USP43 regulates OC cell proliferation and migration. **A** A2780 and TOV-21G cells were transiently transfected with siRNA specific for USP43 (si1 or si2) or control siRNA (NC). **B** CCK-8 assay was employed to study the cell proliferation after silencing USP43 in A2780 and TOV-21G cells (n = 3). **C** Colony-forming assay was employed to investigate the proliferation ability of A2780 and TOV-21G cells after USP43 silencing (n = 3). **D** Cell migration assay was performed in USP43-silenced cells and negative control cells (n = 3)**. E** Overexpression of USP43 was verified using western blotting. **F**, **G** CCK-8 and colony-forming assays showed that USP43 overexpression enhanced proliferation and colony-forming ability of A2780 and TOV-21G cells (n = 3). **H** Transwell assay showed that USP43 overexpression induced the migration of A2780 and TOV-21G cells (n = 3). I, **J** Western blotting confirmed that USP43 influences the expression of migratory proteins such as vimentin, E-cadherin, and N-cadherin. Data are presented as mean ± SD.

To elucidate the role of USP43 in OC pathogenesis, we knocked down USP43 in A2780 and TOV-21G cells and confirmed its knockdown efficiency using western blotting (Fig. 2A). USP43 knockdown significantly inhibited the proliferation and colony formation abilities of A2780 and TOV-21G cells (Fig. 2B, C). Transwell and flow cytometry apoptosis assays indicated that USP43 knockdown inhibited cell migration and promoted apoptosis (Figs. 2D and S2A, B). Furthermore, western blotting showed that silencing USP43 reduced the expression of migration-promoting proteins, such as Vimentin and N-cadherin, and increased the expression of migration-inhibiting proteins, such as E-cadherin (Fig. 2I). Next, we overexpressed USP43 in A2780 and TOV-21G cell lines, and the efficiency was confirmed using western blotting (Fig. 2E). USP43 overexpression promoted the proliferation and colony formation abilities of A2780 and TOV-21G cells (Fig. 2F, G). Simultaneously, USP43 overexpression enhanced cell migration and reduced apoptosis in A2780 and TOV-21G cells (Figs. 2H and S2C, D). Western blotting showed that USP43 overexpression increased the expression of migration-promoting proteins, such as Vimentin and N-cadherin, and reduced the expression of migration-inhibiting proteins, such as E-cadherin (Fig. 2J).

### USP43 regulates ferroptosis in OC

**Fig. 3:**
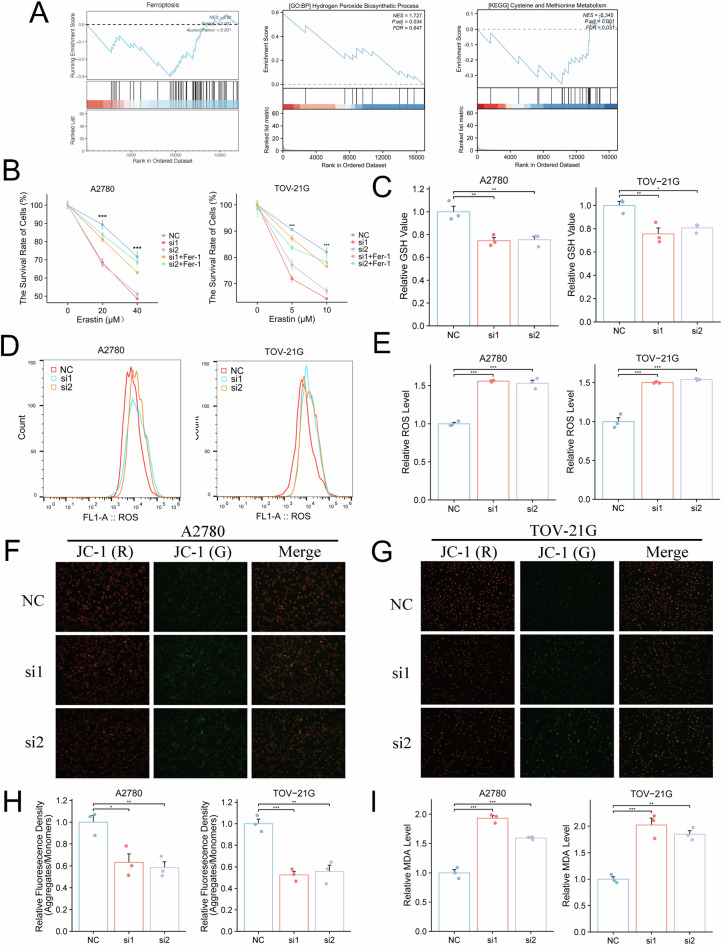
USP43 regulates ferroptosis in OC. **A** Pathways obtained after GSEA following USP43 knockout. **B** Cell viability after different concentrations of erastin and ferrostatin-1 (5 μM) treatment in USP43-depleted and control groups (n = 3). **C** Intracellular glutathione (GSH) level was assessed after erastin (20 μM for A2780 and 5 μM for TOV-21G cells) treatment in USP43-ablation and control groups (n = 3). **D, E** Flow cytometry results and statistical graphs showing the intracellular reactive oxygen species (ROS) levels after treatment with erastin (20 μM for A2780 and 5 μM for TOV-21G cells) in USP43-knockout and control groups (n = 3). **F**–**H** Fluorescence images and statistical graphs showing the intracellular mitochondrial membrane potential after erastin (20 μM for A2780 and 5 μM for TOV-21G cells) treatment (n = 3). **I** Intracellular MDA levels after erastin (20 μM for A2780 and 5 μM for TOV-21G cells) treatment in USP43-knockout and control groups (n = 3). Data are presented as mean ± SD.

To investigate the biological mechanisms by which USP43 regulates OC cells, we performed RNA sequencing following USP43 knockout in A2780 cells. The results revealed significant enrichment of the ferroptosis, peroxisome, and cysteine metabolism pathways (Fig. 3A). The viability of USP43-knockdown OC cells after treatment with different concentrations of the ferroptosis inducer erastin was significantly lower than that of the non-knockdown group, and Ferrostatin-1 (Fer-1) can partially restore the reduced cell activity caused by USP43 knockdown (Fig. 3B). This suggests that USP43 influences the sensitivity of OC cells to ferroptosis inducers. Similarly, upon erastin treatment, USP43-knockdown cells exhibited lower levels of GSH (Fig. 3C), higher production of reactive oxygen species (ROS) (Fig. 3D, E), and lower mitochondrial membrane potential (Fig. 3F–H) than the negative control group, indicating increased oxidative stress in the cells. Additionally, the content of malondialdehyde (MDA), an end product of lipid peroxidation, increased (Fig. 3I). These results demonstrate that silencing USP43 enhances the sensitivity of OC cells to ferroptosis inducers.

### USP43 regulates the transcriptional level of SLC7A11

**Fig. 4:**
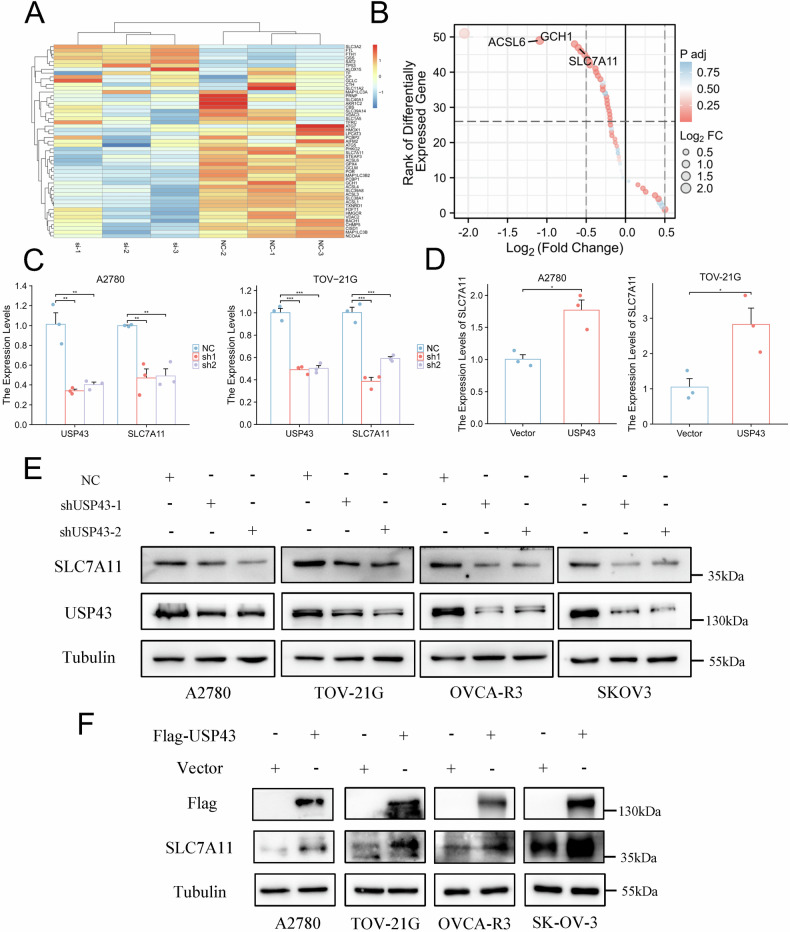
USP43 controls the transcriptional activity of SLC7A11. **A** Heatmap showing changes in ferroptosis-related genes. **B** *ACSL6, GCH1*, and *SLC7A11* were the top three ferroptosis-related genes with the most significant changes. **C**, **D** Knockdown of USP43 reduced *SLC7A11* RNA levels, whereas overexpression of USP43 increased *SLC7A11* RNA levels (n = 3). **E**, **F** Knockdown of USP43 reduced SLC7A11 protein levels, while overexpression of USP43 increased SLC7A11 protein levels. Data are presented as mean ± SD.

To investigate the main targets through which USP43 regulates ferroptosis in OC cells, we analyzed changes in ferroptosis-related genes following USP43 knockdown. Several ferroptosis-related genes were substantially regulated by USP43 (Fig. 4A). By ranking these genes based on fold change, we identified that the top three most affected genes were *ACSL6*, *GCH1*, and *SLC7A11* (Fig. 4B). Through a literature review, we found that *SLC7A11* is a key gene that influences ferroptosis and is a potential drug target. We further validated the regulatory effects of USP43 on *SLC7A11* RNA levels. USP43 knockdown significantly reduced *SLC7A11* RNA levels, whereas its overexpression significantly increased *SLC7A11* RNA levels (Fig. 4C, D). Additionally, we examined the regulation of SLC7A11 protein expression by USP43 across HEK293 cells and four OC cell lines and found that USP43 consistently affected SLC7A11 protein levels in a manner corresponding to the changes in RNA levels (Figs. 4E, F and S3A). As a member of the deubiquitinating enzyme family, USP43 typically functions by binding to other proteins to inhibit their degradation. Therefore, we performed immunoprecipitation experiments to verify whether USP43 binds to SLC7A11, and the results showed that USP43 did not bind to SLC7A11 (Fig. S3B). These findings further support the hypothesis that USP43 influences SLC7A11 expression by regulating its transcription.

### USP43 stablizes FASN by deubiquitination

**Fig. 5:**
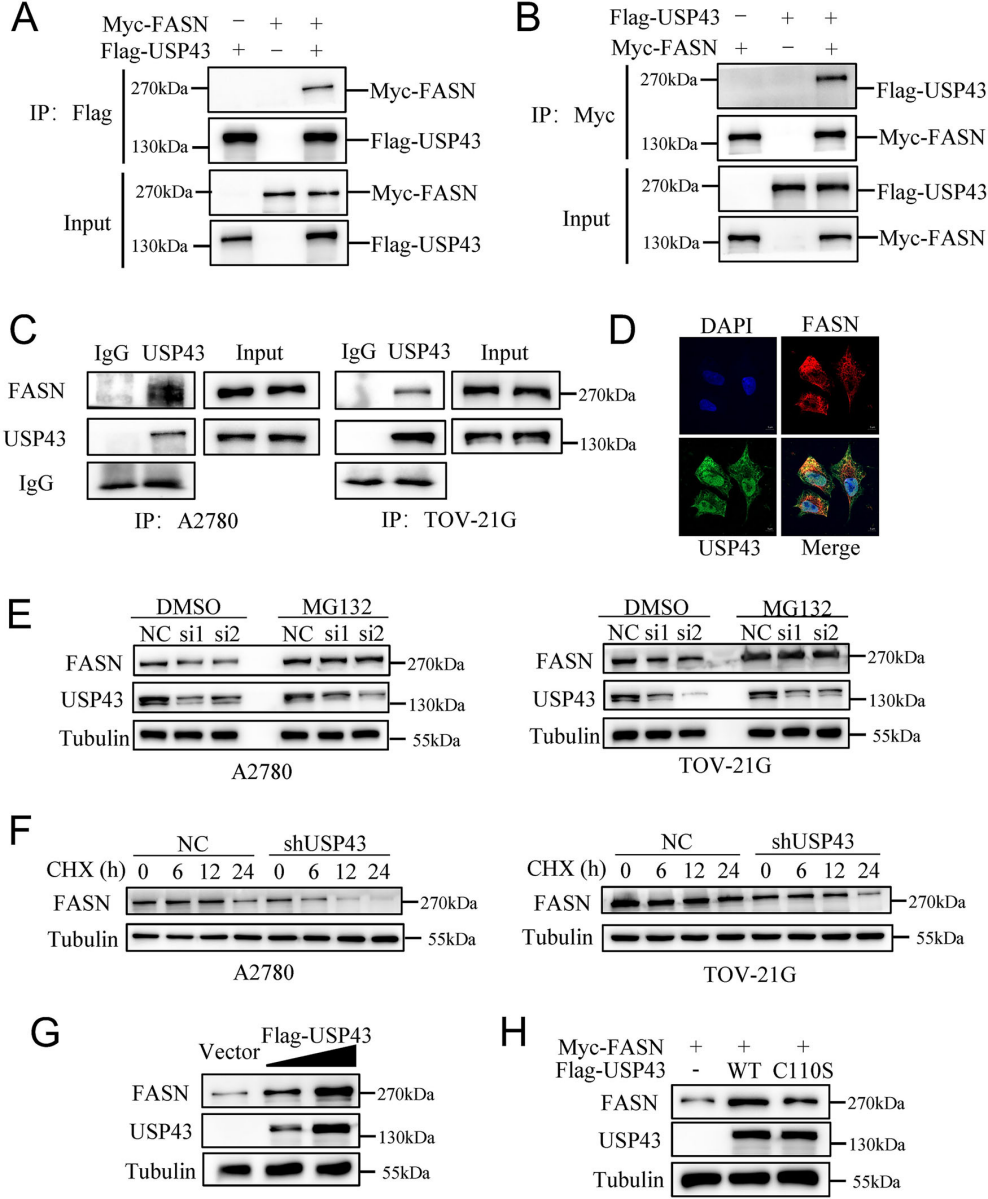
USP43 binds to and attenuates the proteasome-mediated degradation of FASN. **A**, **B** Exogenous immunoprecipitation confirming the interaction between FASN and USP43. **C** Endogenous immunoprecipitation validating the interaction between FASN and USP43. **D** Immunofluorescence showing colocalization of USP43 and FASN. **E** Regulation of FASN protein expression by USP43 in the presence of MG132 (20 μM). **F** Regulation of FASN protein expression by USP43 in the presence of CHX (100 μg/ml). **G** Changes in FASN expression levels based on USP43 levels. **H** Regulation of FASN expression by the C110S mutant of USP43. I Regulation of FASN ubiquitination levels by USP43.

To investigate how USP43 affects the transcription of *SLC7A11*, we conducted IP-MS experiments after overexpressing USP43 in A2780 cells. Based on its unique peptide ranking, FASN was the most abundant protein (Supplementary Table 1). Consequently, we performed an exogenous immunoprecipitation experiment, which demonstrated that USP43 binds to FASN (Fig. 5A, B). We then conducted endogenous immunoprecipitation experiments in both the A2780 and TOV-21G cell lines, confirming that USP43 and FASN interact in OC cells (Fig. 5C). Immunofluorescence assays further showed the colocalization of USP43 and FASN in OC cell lines (Fig. 5D). Thus, USP43 may function by interacting with FASN. Next, we examined whether USP43 affects FASN protein levels. Knockdown of USP43 led to a decrease in FASN protein expression, whereas the addition of the proteasome inhibitor MG132 restored the FASN protein levels (Fig. 5E). Next, protein stability assays were performed. After adding the protein synthesis inhibitor CHX, FASN protein levels in USP43-knockdown cells decreased more significantly over time than those in the control group (Fig. 5F). Additionally, FASN levels increased upon USP43 overexpression in a dose-dependent manner (Fig. 5G). These results indicate that USP43 stabilizes FASN protein expression. To verify whether USP43 regulates FASN through its catalytic domain, we constructed a C110S mutant of USP43 based on the literature. We found that the C110S mutant exhibited a lower ability to stabilize FASN protein levels than wild-type USP43 (Fig. 5H).

**Fig. 6:**
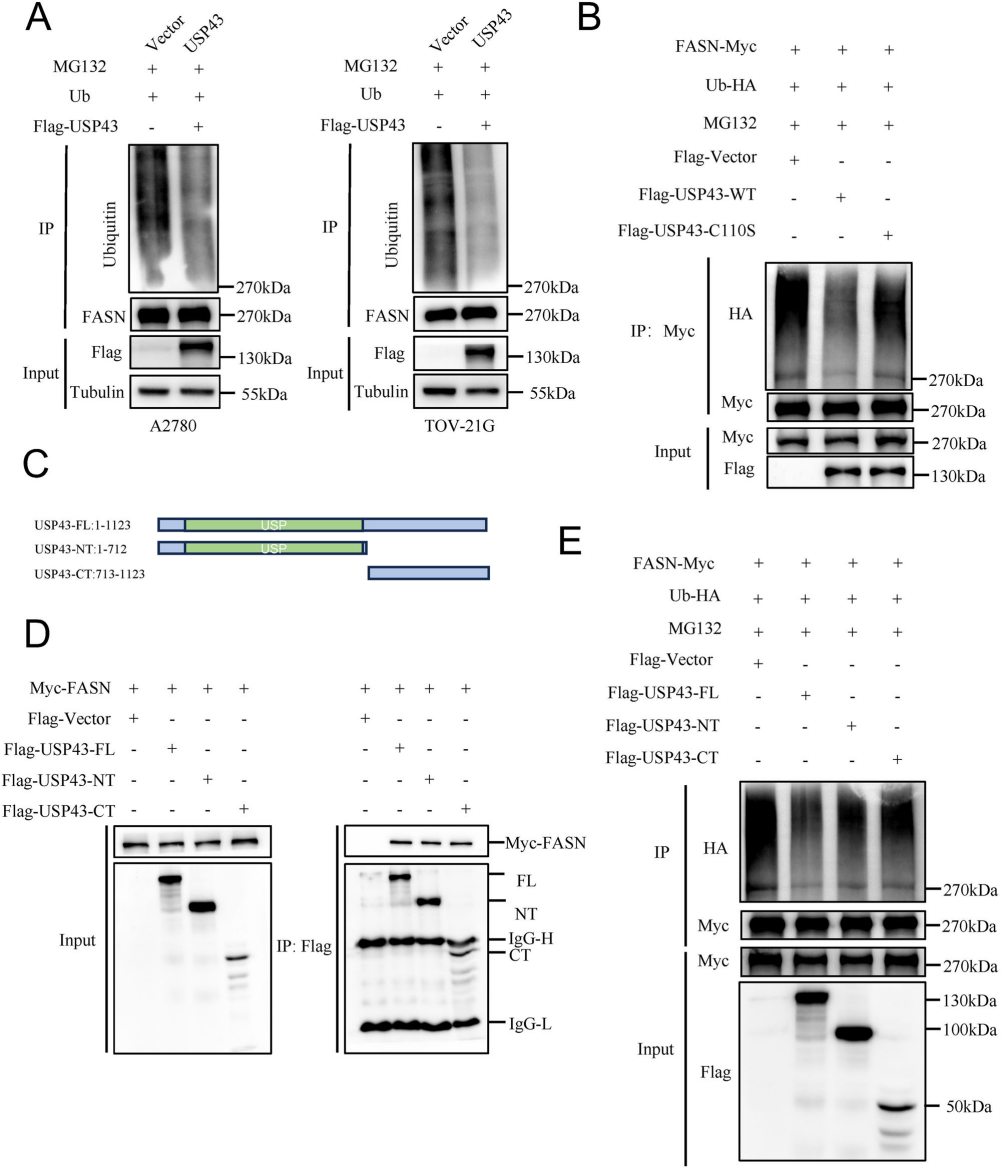
USP43 reduces the ubiquitination level of FASN. **A** Overexpression of USP43 reduces the ubiquitination level of FASN in ovarian cancer cells. **B** C110S mutation abrogates the effect of USP43 on FASN ubiquitination. **C** The schematic diagram illustrating the truncation of full-length USP43. **D** Truncation experiments identifying the specific binding regions of USP43 with FASN. **E** Ubiquitination assays revealed the effect of truncated USP43 on the ubiquitination of FASN.

Further, ubiquitination assays showed that USP43 reduced FASN ubiquitination in ovarian cancer cell lines (Fig. 6A) and The C110S mutant attenuated the ability of USP43 to hydrolyze ubiquitin chains on FASN (Fig. 6B). To identify the interaction sites between USP43 and FASN, we performed truncation experiments by dividing full-length USP43 into N-terminal and C-terminal fragments (Fig. 6C). The results revealed that both the N- and C-terminal regions of USP43 bind to FASN (Fig. 6D). Although both the C- and N-termini of USP43 are capable of binding to FASN, the C-terminus alone exhibits reduced deubiquitinating activity toward FASN compared to the full-length USP43 (Fig. 6E). These findings suggest that USP43 inhibits FASN ubiquitination through its deubiquitinating enzyme activity, thereby reducing proteasomal degradation and stabilizing protein levels.

### USP43 activates SLC7A11 transcription through FASN-HIF1α axis

**Fig. 7:**
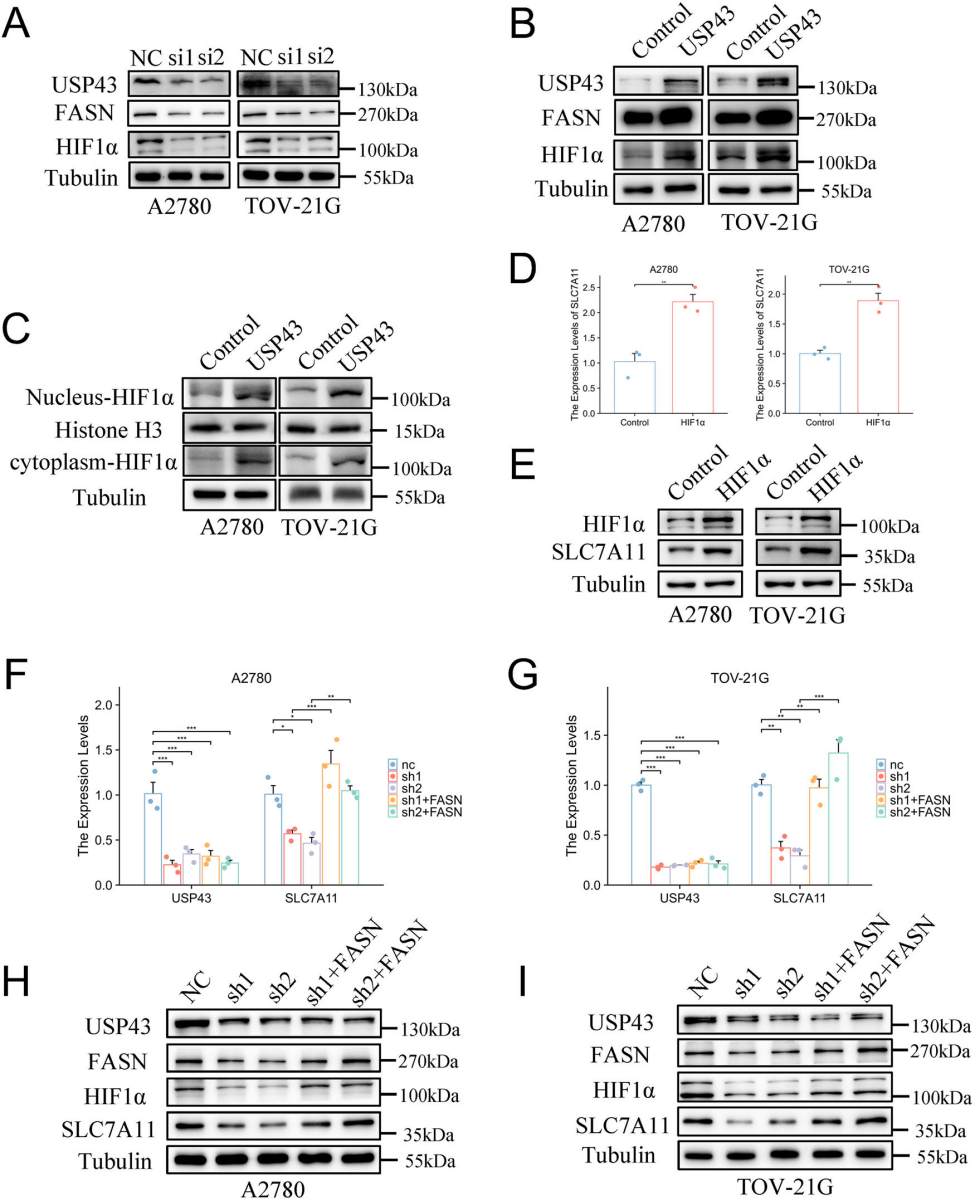
USP43 modifies HIF1α and SLC7A11 by regulating FASN stability. **A**, **B** Western blotting experiments were conducted to estimate the expression levels of FASN and HIF1α after USP43 knockdown and overexpression. **C** We used western blotting to analyze the expression of HIF1α in the cytoplasm and nucleus after USP43 overexpression. **D** Overexpression of HIF1α increases *SLC7A11* mRNA levels (n = 3). **E** Overexpression of HIF1α increases *SLC7A11* protein levels. **F**, **G** The mRNA levels of SLC7A11 assessed using RT-qPCR after FASN was overexpressed in USP43-knockdown cells (n = 3). **H**, **I** The protein levels of HIF1α and SLC7A11 assessed using Western blot after FASN was overexpressed in USP43-knockdown cells. Data are presented as mean ± SD.

To investigate whether USP43 promotes the transcription of SLC7A11 by stabilizing FASN, we reviewed the literature and found that FASN can stabilize HIF1α, and the accumulation of HIF1α in the nucleus subsequently promotes the transcription of SLC7A11 [30]. We performed Western Blot (WB) analysis in A2780 and TOV-21G cell lines. Knockdown of USP43 reduced FASN and HIF1α levels (Fig. 7A), whereas overexpression of USP43 led to increased expression of FASN and HIF1α (Fig. 7B). Furthermore, overexpression of USP43 increased the expression of HIF1α in both the nucleus and cytoplasm (Fig. 7C). Meanwhile, overexpression of HIF1α upregulated the mRNA levels and the protein levels of *SLC7A11* in A2780 and TOV-21G cells (Fig. 7D, E). Thus, USP43 may activate *SLC7A11* transcription via the FASN-HIF1α axis. To further confirm this hypothesis, we overexpressed FASN in USP43-knockdown cells and observed that FASN restored the mRNA levels of SLC7A11 reduced by USP43-knockdown (Fig. 7F, G) and upregulated the protein expression levels of HIF1α and SLC7A11(Fig. 7H, I). These results indicate that USP43 promotes the transcriptional activation of *SLC7A11* by stabilizing FASN and subsequently driving the nuclear accumulation of HIF1α. Next, we examined whether FASN could rescue the effects of USP43 on ferroptosis in OC. Overexpressing FASN in USP43-knockdown OC cells could reverse the consequent reduction in GSH (Fig. S4A) and increase in MDA levels (Fig. S4B). These results indicate that USP43 regulates the ferroptosis process in OC cells through FASN-HIF1α axis.

Platinum resistance is a major contributor to the poor prognosis of OC, and USP43 influences the sensitivity of tumor cells to platinum-based drugs. To confirm whether SLC7A11 can counteract the effects of USP43 on the malignant phenotype of OC cells and provide new options for clinical treatment, we established the platinum-resistant cell lines A2780-DDP and SKOV3-DDP and confirmed their resistance (Fig. S5A). Western blotting demonstrated that SLC7A11 expression was significantly higher in resistant cells than in the parental cells (Fig. S5B). Subsequently, we knocked down USP43 while overexpressing SLC7A11 in the resistant cells (Fig. S5C, D). USP43 knockdown significantly inhibited the proliferation and colony formation ability of resistant OC cells, whereas SLC7A11 overexpression reversed this effect (Fig. S6A, B). Similarly, flow cytometry apoptosis and Transwell migration assays showed that USP43 knockdown promoted apoptosis and inhibited cell migration, whereas SLC7A11 overexpression reversed these phenotypes (Fig. S6C–F). Thus, USP43 modifies the malignant phenotype of OC cells in an SLC7A11-dependent manner.

### Targeting SLC7A11 in tumors with high USP43 expression can benefit cisplatin-mediated inhibition of tumor growth

**Fig. 8:**
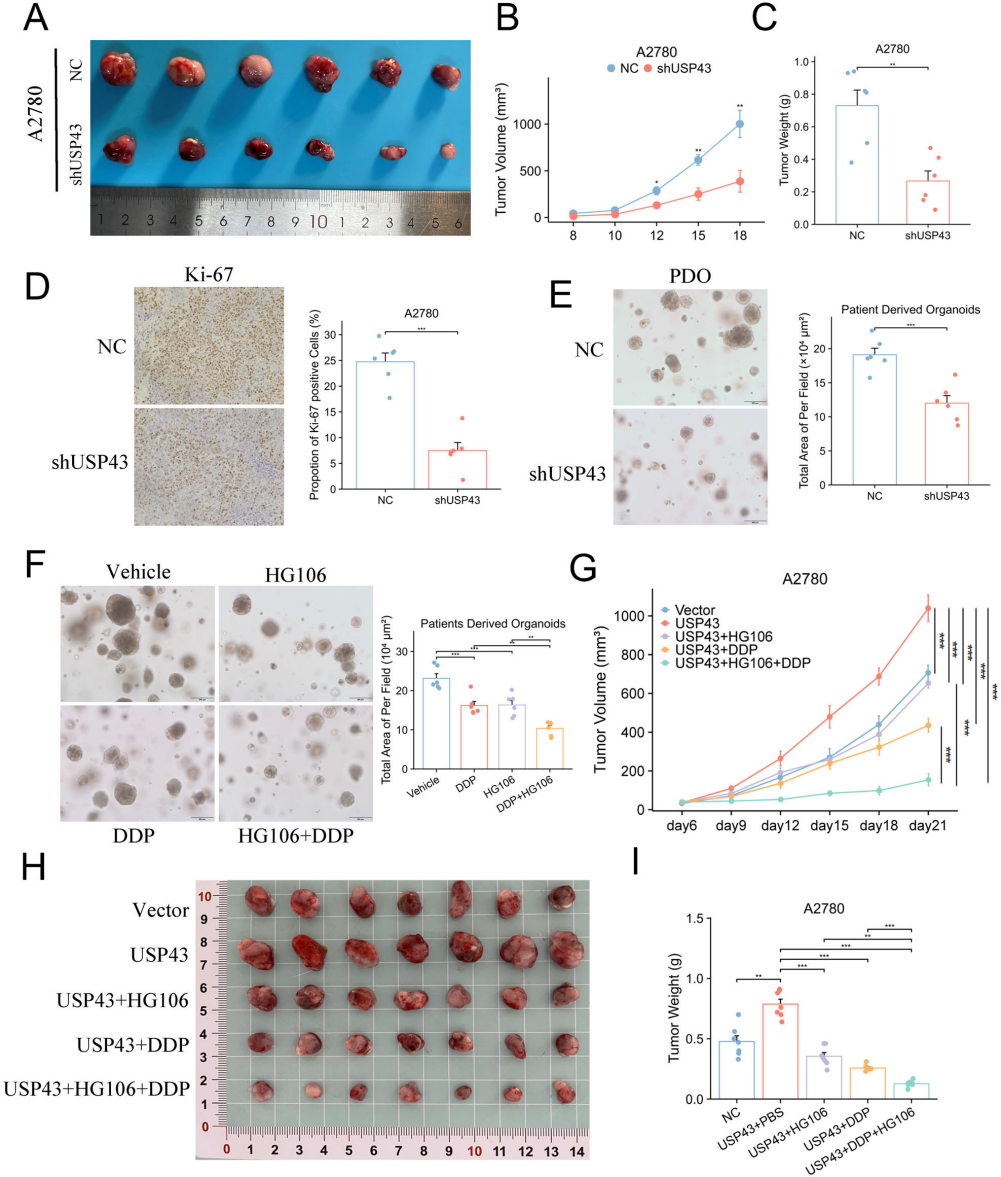
In vivo experiments validating the impact of USP43 on the tumorigenic capacity and the combined effect of SLC7A11 inhibitor with cisplatin. **A**–**C** Tumor volumes, growth curves, and tumor weights of A2780-derived xenografts in nude mice from the USP43-knockdown and control groups. n = 6/group. **D** Representative immunohistochemistry images and statistical graphs of Ki67 levels in the control and USP43-knockdown groups. n = 6/group. **E** Representative images and statistical graphs of PDOs in the control and USP43-knockdown groups. n = 6/group. **F** Representative images and statistical graphs of PDOs after treatment with HG106 and cisplatin, both individually and in combination. n = 6/group. **G**–**I** Tumor volumes, weights, and growth curves of xenografts derived from A2780 cells overexpressing USP43 after treatment with HG106 and cisplatin, both individually and in combination. n = 7/group. Data are presented as mean ± SD.

To validate the effect of USP43 on the tumorigenic capacity of OC cells in vivo, we performed a subcutaneous xenograft tumor assay in nude mice. USP43 knockdown significantly inhibited the tumorigenic capacity of OC cells, resulting in slower tumor growth and significantly lower tumor volume and weight than in the control group (Figs. 8A–C and S7A). IHC experiments showed that Ki67 levels in the USP43-knockdown group were significantly lower than those in the control group (Fig. 8D). In order to verify the effectiveness of USP43 in an environment close to real human OC tissues, we constructed an ovarian cancer patients derived organoids (PDOs) model. After infection with shUSP43 virus, the growth of PDOs slowed down, and the size of PDOs were significantly smaller than those of the control group (Fig. 8E). These results further confirm the critical role of USP43 in promoting tumor formation. HG106 is a selective SLC7A11 inhibitor. To verify whether HG106 can act synergistically with cisplatin to inhibit tumors, we performed combination treatment experiments using both PDOs and USP43-overexpressing OC cell derived xenografts. The results demonstrated that both cisplatin and HG106 alone could inhibit tumor growth, and their combination further synergistically enhanced tumor cell death (Fig. 8F–I and S7B).

### YY1 promotes the expression of USP43

**Fig. 9:**
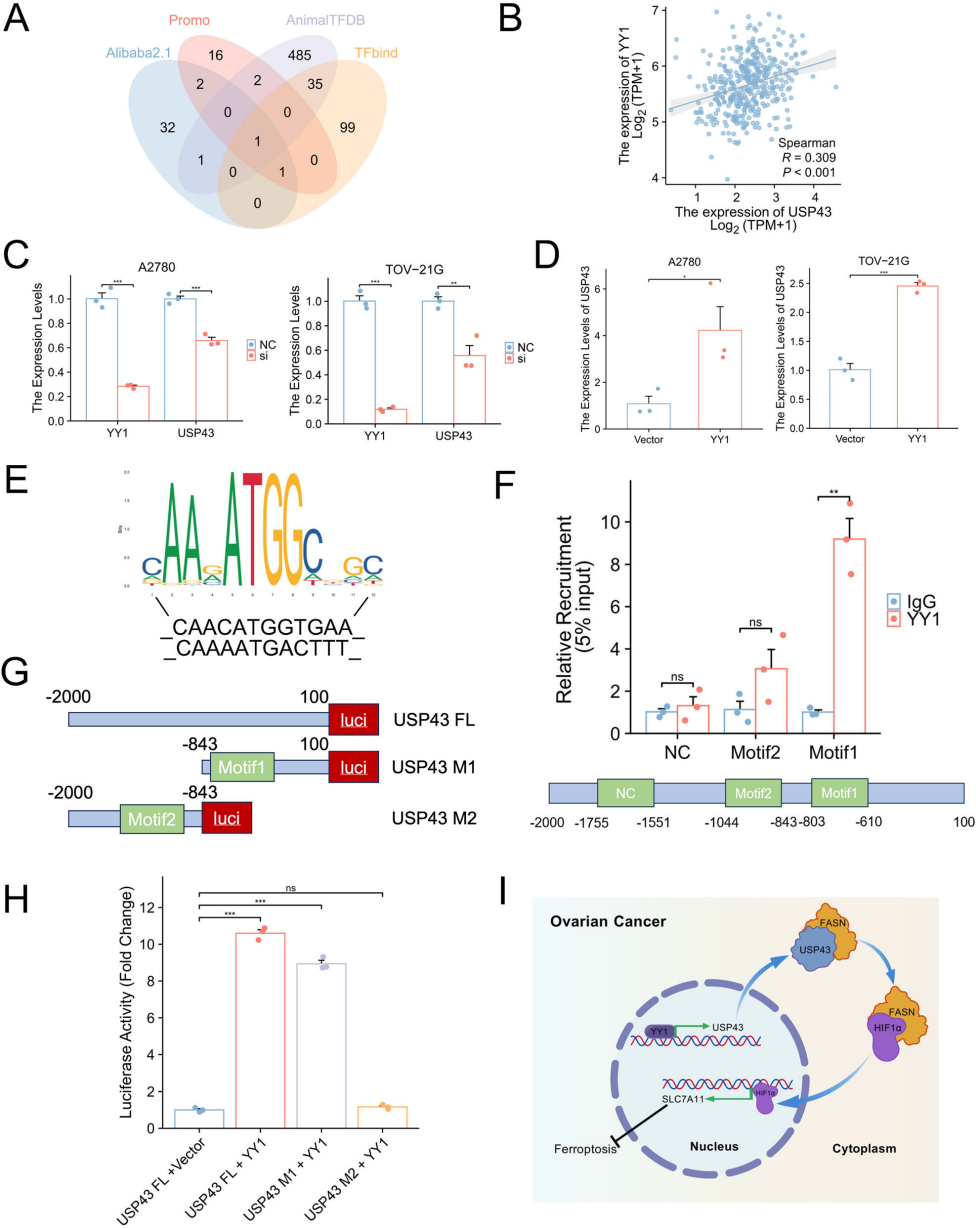
YY1 enhances the transcriptional expression of USP43. **A** Common transcription factors of USP43 predicted using four databases. **B** Correlation analysis between YY1 and USP43 expression levels in OC using TCGA. **C** Overexpression of YY1 increases *USP43* mRNA levels (n = 3). **D** Knockdown of YY1 decreases *USP43* mRNA levels. **E** JASPAR prediction of YY1-binding motif regions (n = 3). **F** ChIP-qPCR validation of YY1 bound to the predicted USP43 promoter region (n = 3). **G** Construction of full-length USP43 promoter sequence and truncations. **H** Validation of YY1 binding to the USP43 promoter region using luciferase assay (n = 3). **I** A simplified working model depicting YY1-activated USP43, which facilitates ferroptosis suppression in OC cells by inducing *SLC7A11* transcription. USP43 binds to and stabilizes FASN via its deubiquitinating enzyme activity. Subsequently, the stabilized FASN maintains HIF1α levels, promoting its accumulation in the nucleus. Finally, nuclear HIF1α enhances the transcription of *SLC7A11*, aiding OC cells in resisting ferroptosis. Data are presented as mean ± SD.

Transcriptional activation is a key mechanism underlying the upregulation of oncogene expression. To identify transcription factors promoting USP43 expression in ovarian cancer, we used the Alibaba2.1, Promo, AnimalTFDB, and TFbind databases to predict potential transcription factors binding to the USP43 promoter. Surprisingly, only transcription factor YY1 was predicted in all four databases (Fig. 9A). Subsequently, analysis of TCGA data revealed a significant positive correlation between YY1 and USP43 expression (Fig. 9B). First, we validated the knockdown and overexpression efficiency of YY1 in A2780 and TOV-21G cell lines using western blotting (Fig. S8). Through qPCR experiments, we confirmed that the overexpression of YY1 enhanced the transcription of USP43, whereas YY1 knockdown reduced USP43 transcription (Fig. 9C, D). Using the JASPAR database, we predicted two potential YY1-binding domains in the USP43 promoter (Fig. 9E). Based on the locations of these two domains, we designed specific qPCR primers and conducted a chromatin immunoprecipitation (ChIP) assay (Fig. 9F). Furthermore, we constructed USP43 promoter truncations and performed dual-luciferase reporter assays (Fig. 9G, H). Both the ChIP and luciferase assays showed that YY1 promotes USP43 transcription by binding to the M1 domain.

## Discussion

By family screening, our study shows that USP43 not only has significant diagnostic value but also serves as an important prognostic marker for patients with OC. The present study identified the role and potential molecular mechanism of USP43 in OC cell ferroptosis. Reducing the expression of USP43 can inhibit the proliferation and migration of both epithelial and drug-resistant OC cells and suppress tumor growth in vivo. Mechanistically, USP43 activates *SLC7A11* expression by stabilizing FASN and promoting nuclear accumulation of HIF1α. Thus, USP43 is a promising therapeutic target for OC, and the induction of ferroptosis by inhibiting *SLC7A11* in combination with cisplatin is a good choice for OC patients.

Although some studies have revealed that members of the deubiquitinating enzyme USP family play extensive and critical roles in tumorigenesis, exhibiting either oncogenic or tumor-suppressive functions, the existing research remains incomplete given the considerable size and diversity of this large gene family [31]. USP43 remains one of the least characterized members within this family. Emerging evidence indicates its functional involvement in multiple biological processes and malignancies. For example, USP43 is responsible for activating HIF response by increasing the nuclear accumulation of HIF1α [32]. While in malignancies, USP43 drives the proliferation and metastasis of colorectal cancer and cervical cancer [19, 33]. In bladder cancer, USP43 promotes glycolysis by regulating C-myc transcriptional activity [18]. Although these investigations have delineated USP43’s regulatory roles in tumor proliferation, metastasis, and glycolytic reprogramming, its functional engagement with other cellular processes—particularly the modulation of cell death modalities—has yet to be systematically explored. Current evidence is restricted to a solitary publication demonstrating USP43-mediated apoptotic regulation in ovarian cancer via the Wnt/β-catenin signaling axis [34], while its potential involvement in governing ferroptosis, necroptosis, and immunogenic cell death remains uncharted in oncological research.

Ferroptosis is a form of non-apoptotic programmed cell death that is regarded as a promising therapeutic target for OC. In ascites-derived OC cells, the fatty acid desaturases SCD1/FADS2 regulated the equilibrium of redox-driven ferroptosis and lipid metabolic activity. Subsequently, a combination therapy involving cisplatin and SCD1/FADS2 inhibitors synergistically inhibited OC cell dissemination, presenting a promising chemotherapeutic strategy for managing peritoneal metastases in epithelial ovarian cancer [35]. Another protein, MEX3A, suppressed ferroptosis in p53 wild-type OC. By reducing the stability of wild-type p53, MEX3A facilities OC progression [36]. Additionally, PARPi has shown better anticancer efficacy in patients with OC harboring BRCA mutations than in persons without BRCA mutations and promotes ferroptosis by inhibiting the expression of SLC7A11 and suppress the progression of BRCA-positive OC when combined with ferroptosis inducers [37]. In our study, we have demonstrated that USP43 is a novel regulated target affecting ferroptosis in OC, providing a new option for targeting ferroptosis pathways to treat ovarian cancer.

To delineate the molecular circuitry underlying USP43-mediated ferroptosis regulation, we employed immunoprecipitation coupled with mass spectrometry (IP-MS) profiling. The proteomic profiling data demonstrated that FASN exhibited the highest abundance of uniquely mapped peptide fragments, establishing it as the top-ranked candidate in the IP-MS interactome analysis. Additionally, co-immunoprecipitation assays confirmed a direct physical interaction between USP43 and FASN. FASN serves as a master regulator of cellular lipid homeostasis, orchestrating de novo lipogenesis through its enzymatic synthesis of long-chain fatty acids. Upregulation of FASN promotes cancer progression and is associated with poor prognosis in patients with multiple tumors [38]. Post-translational modifications (PTMs) are biochemical changes that occur after protein synthesis and play key roles in regulating protein activity in response to cellular signals and cancer development. These post-translational modifications can regulate the enzymatic activity, stability, and role of FASN in tumor metabolism. For example, FBXW7β acts as an E3 ligase of FASN, inhibiting colorectal cancer growth by degrading FASN [39]. Additionally, FABP5 interacts with FASN, triggering the activation of the ubiquitin-proteasome pathway, which leads to a reduction in FASN expression and lipid accumulation. This inhibits mTOR signaling and stimulates autophagy [40]. Furthermore, USP22 deubiquitinates and stabilizes FASN. After suppression by hydrogen peroxide (H2O2)-induced p53 expression, the downregulation of USP22 leads to decreased stabilization of FASN, thereby hindering fatty acid synthesis in p53 wild-type colorectal cancer cells [41]. Emerging evidence further delineates FASN’s pleiotropic mechanisms in ferroptosis modulation. Genetic ablation of FASN disrupts the homeostatic ratio of saturated to unsaturated fatty acids, priming cells for ferroptosis vulnerability [42]. Paradoxically, in sorafenib-resistant hepatocellular carcinoma (HCC) models, FASN orchestrates nuclear accumulation of HIF1α to drive SLC7A11 transactivation, thereby conferring ferroptosis resistance [30]. While these mechanistic insights illuminate the regulatory capacity of FASN in ferroptosis, it remains elusive whether USP43 orchestrates ferroptotic pathways in ovarian malignancies through FASN-dependent cascades, nor are its precise molecular targets within ferroptosis fully characterized.

SLC7A11 functions as a master negative regulator of ferroptosis. It effectively blocks ferroptosis cascade activation through maintaining intracellular glutathione (GSH) homeostasis and amplifying antioxidant defense systems. Across diverse tumor entities, the transcriptional upregulation of SLC7A11 confers robust protection against oxidative insults, driving both malignant cell survival and therapeutic resistance. This dual role in redox balancing and stress adaptation positions SLC7A11 as a high-priority molecular target for innovative anticancer modalities. In our study, RNA-seq analysis of USP43-knockdown OC cells revealed enrichment of the ferroptosis signaling pathway, with *SLC7A11* among the top three genes with the highest fold. Subsequently, we demonstrated that USP43 regulates *SLC7A11* expression at both the RNA and protein levels. Various proteins influence ferroptosis by regulating SLC7A11. The GCN2-eIF2α signaling axis activates SLC7A11 transcription via ATF4 under amino acid-depleted conditions [43]. Conversely, tumor suppressors, including p53 and ATF3, function as transcriptional repressors of SLC7A11 [44, 45]. In hepatocellular carcinoma, DNA damage-induced ATR kinase activation phosphorylates and stabilizes USP20, which subsequently stabilizes SLC7A11 by exercising its deubiquitinating enzyme activity to disassemble K48-linked polyubiquitination at lysine residues K30/K37, thereby counteracting its proteasomal targeting [46]. Considering that USP43 does not directly bind to SLC7A11, we hypothesize that USP43 regulates SLC7A11 expression via FASN. Because FASN can interact with HIF1α and activate *SLC7A11* transcription, we subsequently employed rescue experiments to confirm that USP43 indeed regulates HIF1α and SLC7A11 through FASN. The results showed that FASN is required for USP43 to regulate the expression levels of HIF1α and SLC7A11, which precisely explains the mechanism by which USP43 regulates SLC7A11. Our study provides inaugural evidence that USP43 orchestrates ferroptosis susceptibility in ovarian cancer, mechanistically delineating its governance of the FASN-HIF1α-SLC7A11 signaling axis. Crucially, we propose the therapeutic paradigm of rational combination therapy, demonstrating that USP43-high neoplasms exhibit enhanced vulnerability to synergistic tumoricidal efficacy when targeted with SLC7A11 inhibitors in concert with platinum-based agents like cisplatin.

The upregulation of oncogene in cancer is largely rooted in transcriptional control. Through integrated computational prediction and experimental validation of upstream regulatory networks, we identified YY1 (Yin Yang 1) as a master transcriptional activator that directly binds to the USP43 promoter, driving its aberrant expression. YY1, a multifunctional protein belonging to the GLI-Krüppel family, is a zinc-finger protein that plays a crucial role in gene expression regulation in various biological processes, including development, differentiation, and cellular responses to external stimuli [47]. Moreover, this zinc-finger protein acts as both an activator and repressor of transcription, depending on the context and specific target genes involved [48]. In OC, YY1 overexpression is associated with poor patient outcomes [49]. In our study, YY1 was identified as the primary factor responsible for the dysregulation of USP43 in OC, and its expression positively correlated with that of USP43. This suggests that YY1 may affect the prognosis of ovarian cancer patients through USP43. Interestingly, YY1 and YY2 have been verified to regulate ferroptosis by competing for the promoter region of SLC7A11, thereby influencing its expression [50]. Further research is required to explore how YY1 and HIF1α coordinate to activate SLC7A11 expression and the role that USP43 played in it.

Together, these results revealed that USP43 orchestrates ferroptosis by stabilizing the FASN protein and eventually activating *SLC7A11* expression, highlighting the role of USP43 regulation at the post-translational level in OC progression (Fig. 9I). According to our results, USP43 is specifically overexpressed in malignant epithelial cells, with minimal expression in other cell types. This suggests that USP43 may serve as a promising therapeutic target for ovarian cancer, potentially offering efficacy with limited off-target effects. However, a major challenge in targeting deubiquitinating enzymes lies in the structural similarities shared among members of the USP family, particularly within their conserved catalytic domains. This structural homology makes it difficult to develop highly selective inhibitors. Inhibitors with poor specificity may simultaneously suppress the functions of multiple USP family members, leading to unintended consequences or even opposing effects. Such potential risks must be carefully considered when developing USP43-targeted inhibitors. Nonetheless, the future development of highly specific USP43 inhibitors could offer a novel, safe, and effective therapeutic strategy for patients with ovarian cancer.

## Conclusions

We characterized the ferroptosis inhibition function of the USP family member USP43 in OC. Gain- and loss-of-function assays demonstrated that USP43 promotes a malignant phenotype and resistance to ferroptosis in OC cells. Mechanistically, USP43 is transcriptionally activated by YY1 and stabilizes FASN by inhibiting its ubiquitination and degradation. The stabilized FASN subsequently stabilizes HIF1α and activates the expression of *SLC7A11*. Collectively, our study revealed a novel molecular event underlying ferroptosis, in which USP43 induces FASN expression at the post-translational level, activates *SLC7A11* transcription, and drives OC progression, suggesting that USP43 may be a potential target for OC therapy.

## Methods and materials

### Data acquisition

Bulk RNA-seq data and clinical information for the OC cohort from TCGA and GTEx were downloaded from the UCSC Xena browser (https://xena.ucsc.edu/). scRNA-seq data for OC and matched non-malignant ovarian samples (GSE184880) were downloaded from the Gene Expression Omnibus (GEO) database (https://www.ncbi.nlm.nih.gov/geo/). All data acquisition and subsequent analyses were performed using open-source R version 4.2.2. The pROC package and clusterProfiler package were employed to generate ROC curve and GSEA analysis.

### scRNA-seq analysis

The R package Seurat was used to process and analyze scRNA-seq data. Cell quality control and filtering were performed to exclude cells that (1) expressed genes in fewer than three cells, (2) contained fewer than 300 genes, or (3) expressed more than 50% of mitochondrial genes. Standard analysis protocol was employed, followed by dimension reduction and clustering. The R package Harmony was used to adjust for batch effects, and specific markers of various cell types were used to define different cell subsets. Cell types were annotated based on cell-type specific markers.

### Reagents and antibodies

MG132 (#HY-13259) was purchased from MedChemExpress (Shanghai, China). Erastin (#S7242) was purchased from Selleck Chemicals (Shanghai, China). Ferrostatin-1 (HY-100579) was purchased from MedChemExpress (Shanghai, China). Cell counting kit-8 (CCK-8, #CK04) was purchased from Dojindo Laboratories (Tokyo, Japan). HG106 (#T67839), a SLC7A11 inhibitor, was purchased from TOPSCIENCE (Shanghai, China).

The antibodies against USP43 (sc-393895; western blotting, co-immunoprecipitation) was purchased from SANTA CRUZ Biotechnology (Dallas, TX, USA). SLC7A11/xCT (A2413; western blotting), FASN (A19050; western blotting), and YY1 (A19569; ChIP) were purchased from ABcolonal (Wuhan, China). USP43(AP14283b; IHC) was obtained from ABCEPTA Biotechnology (Suzhou, China). HIF1α (20960-1-AP; western blotting), Tubulin (11224-1-AP; western blotting), MYC-tag (60003-2-Ig; western blotting and co-immunoprecipitation), ubiquitin (10201-2-AP; western blotting), IgG (SA00001-2; western blotting), and Ki-67 (27309-1-AP; IHC) were procured from Proteintech (Wuhan, China). Flag-tag (F1804; western blotting and co-immunoprecipitation) were purchased from Sigma-Aldrich (St Louis, MO, USA).

### Cancer cell lines and treatment

The human OC cell lines TOV-21G, A2780, OVCA-R3, and SKOV3, and the human embryonic kidney cell line HEK293T were obtained from ATCC and cultured in Dulbecco’s modified Eagle medium (DMEM) in an incubator (37 °C, 5% CO_2_). Fetal bovine serum (10%) and penicillin-streptomycin (10,000 U/mL) were added to the DMEM before culturing. All cell lines were authenticated by short tandem repeat (STR) analysis and confirmed to be free of mycoplasma contamination. Cells within 8 passages after resuscitation were used for experiments.

### Human tumor samples

The study design was reviewed and approved by the Human Research Ethics Committee of the Fudan University Shanghai Cancer Center (FUSCC). A total of 165 cancer and 74 normal ovarian epithelial tissues were collected following surgery and embedded in paraffin. Written informed consent was obtained from all patients. Tissue microarrays (TMAs) were prepared for subsequent IHC analysis.

### IHC

Paraffin-embedded tumor or normal tissue sections were dewaxed in pure xylene and rehydrated in alcohol at gradually decreasing concentrations. After antigen retrieval using 1× Citrate-EDTA Antigen Retrieval Solution (Beyotime) via microwave heating, the slices were incubated in 3% hydrogen peroxide and goat serum sequentially at room temperature for 15 min. Sections were incubated overnight with primary antibodies against USP43 and Ki67 at 4 °C, followed by secondary antibody incubation with goat anti-rabbit/mouse IgG at room temperature. Immunodetection was performed using 3,3′-diaminobenzidine (DAB), counterstained with hematoxylin. Finally, the brown color intensity and proportion of positive cells were determined using an optical microscope by two experienced pathologists. The staining intensity of each section was graded as 0 (no detectable staining), 1 (weak), 22 (moderate), or 3 (strong). Concurrently, the percentage of positively stained tumor cells of each section was quantified using the following criteria: 1 (<10%), 2 (11–50%), 3 (51–75%), and 4 (>75%). The final Immunoreactive Score (IRS) was calculated by multiplying the intensity score by the percentage score.

### Cell transfection and stable cell line construction

Human full-length *USP43, FASN*, and *SLC7A11* sequences were synthesized and used for overexpression assays. Small interfering RNAs (siRNAs) targeting USP43 were designed for knockdown assays. Plasmids and siRNAs were introduced into cells with empty plasmids or negative sequences as controls. Lipofectamine 2000 transfection reagent and PEI were used for transfection following the manufacturer’s instructions. To generate stable cell lines, the target plasmid and packaging plasmids were co-transfected into 293T cells, and the virus solution was collected 48 h later. The cells were then infected with the viral solution and screened for antibiotics.

### Cell viability

The proliferation of A2780 and TOV-21G cells was measured using a CCK-8 assay after drug treatment or transfection. In short, cells were diluted (2500 cells per 100 μL) and seeded into 96-well plate. After incubation with the CCK-8 reagent for 2 h, the absorbance of each well was measured at 450 nm.

### Colony formation assay

A2780 and TOV-21G cells were diluted (1000 cells per well) and seeded into 6-well plates. The cells were cultured for approximately half a month. The cells were washed with PBS and fixed with methanol. Subsequently, crystal violet (0.1%) was used to stain the colonies. The visible colonies were photographed and counted.

### Transwell assay

Transwell assays were performed using Transwell insert chambers and 24-well plates. The cells were resuspended in a serum-free medium and seeded into the upper chambers. Medium (600 μL) with fetal bovine serum (10%) was added into lower chambers. After 24 h, migrating cells were washed with PBS and fixed with methanol for 15 min. The migratory cells were stained with crystal violet (0.1%) and counted.

### Apoptosis assay

The A2780 and TOV-21G cells were treated for 48 h, digested, and collected. The Annexin V-PE/7-AAD Apoptosis Detection Kit (Vazyme, China) was used to perform the apoptosis assay following the manufacturer’s instructions. Cells were washed and resuspended in binding buffer. Next, Annexin V-PE and 7-AAD were sequentially added to the cell suspensions and incubated for 10 min on ice in the dark. Flow cytometry was performed to analyze the rate of apoptosis.

### Western blotting and antibody

FASN stabilizes HIF1α, and the accumulation of HIF1α in the nucleus subsequently promotes the transcription of SLC7A11. Hence, to investigate whether USP43 promotes the transcription of SLC7A11 by stabilizing FASN, we performed western blotting. RIPA mixed with protease inhibitors was used to lyse the targeted cells and obtain total proteins. After quantification using a bicinchoninic acid assay kit, specific amount of protein was subjected to SDS-PAGE. The separated proteins were then transferred onto PVDF membranes and blocked with non-fat milk (5%). The indicated primary antibodies and corresponding secondary antibodies were incubated with the PVDF membranes. Finally, autoradiography was performed to visualize the proteins using an ECL detection system.

### Immunoprecipitation (IP)

Briefly, cell lysates were prepared from A2780 and TOV-21G cells using lysis buffer. The lysates were then incubated with primary antibody against the target protein overnight at 4 °C with gentle rotation. Protein G beads were added to the lysates and incubated for an additional 2 h at 4 °C. The beads were then washed at least six times with wash buffer to remove non-specifically bound proteins. The immunoprecipitated protein complexes were eluted from the beads by boiling in 2× loading buffer, and the eluted proteins were separated by SDS-PAGE and transferred onto a PVDF membrane for immunoblotting. Mass spectrometry (MS) was used to detect possible interaction protein.

### Immunofluorescence staining

A2780 and TOV-21G cells were plated on fibronectin-coated glass coverslips. The cells were fixed with paraformaldehyde (4%) for 15 min and permeabilized with Triton X-100 (0.3%) for 10 min at room temperature. After blocking with goat serum (10%) for 1 h, the cells were incubated overnight with anti-USP43 and anti-FASN antibodies. The next day, the cells were incubated with the corresponding secondary antibodies for 1 h, and the stained cells were observed by confocal microscopy.

### In vivo ubiquitination assay

After transfection with the indicated plasmids for 48 h, MG132 (20 μM) was added into corresponding cells. The cells were incubated in lysis buffer containing protease inhibitor for 1 h and centrifuged. The supernatant was collected and incubated with the indicated antibodies and protein A/G magnetic beads. After washing the magnetic beads several times, the proteins pulled down by the antibody were boiled and subjected to SDS-PAGE and immunoblotting.

### RNA extraction and real-time quantitative PCR

Total RNA was isolated from the relevant cell samples using RNAiso Plus (Takara, Japan), in accordance with the manufacturer’s instructions. Total RNA (500 ng) from each cell sample was reverse-transcribed into complementary DNA using Hiscript III qRT SuperMix (Vazyme, China). The relative mRNA expression was calculated using the 2^−ΔΔCT^ formula and normalized with β-actin as the reference gene. Primers for qPCR were synthesized at Tsingke Biotech Co. (Beijing, China) and are listed in Table S1.

### ROS, MDA, and GSH level estimation

After erastin treatment in the indicated cells, the intracellular levels of ROS, MDA, and GSH were determined. Total ROS production was measured using an ROS detection kit (Njjcbio, Nanjing, China). DFC signals were detected with emission at 525 nm and excitation at 488 nm. The relative concentration of intracellular MDA in the treated cells was determined using an MDA detection kit (DOJINDO, Shanghai). Fluorescence signals were detected with emission at 540 nm and excitation at 590 nm. GSH levels in the treated cells were detected using a GSH assay kit (Beyotime, China) following the manufacturer’s instructions. Absorbance was measured at an optical density of 412 nm.

### Mitochondrial membrane potential

Indicated A2780 and TOV-21G cells were seeded in 6-well plates. After corresponding treatment, JC-1 working solution was added and incubated for 20 min at 37 °C. The supernatant was aspirated, and the cells were washed twice with JC-1 staining buffer. The ratio of aggregates to monomers was calculated using a fluorescence microscope.

### ChIP

ChIP assays were performed using a Sonication ChIP Kit (Abclonal, China), following the manufacturer’s instructions. After crosslinking the target samples with 1% formaldehyde, cells were lysed and sonicated. The control IgG and anti-YY1 antibodies were added to the samples and incubated overnight for immunoprecipitation experiments. After adding the elution buffer for reverse crosslinking, the DNA was purified for qPCR analysis. The primers used to detect promoter occupancy of USP43 are listed in Table S1.

### Dual-luciferase reporter assay

The human USP43 promoter sequence, spanning from −2000 bp to +100 bp of the transcription start site and its truncated sequences, were PCR amplified and cloned into pGL3 basic luciferase vector. These USP43 promoter luciferase reporter constructs and Renilla luciferase reporter constructs were co-transfected into 293 T cells based on experimental design. After 48 h, the Dual-Luciferase Assay System (Yeasen, China) was used to determine firefly and Renilla luciferase activities according to the manufacturer’s protocol. The Alibaba2.1, Promo, AnimalTFDB, and TFbind databases were used to predict potential transcription factors.

### Patient derived organoids establishment and culture

The construction and culture of OC-derived organoids are all based on the manufacturer’s instructions (D1 Medical Technology, Shanghai, China). Simply, fresh tissue from ovarian cancer patients were digested in tissue digestion solution for 1 h after cleaning with PBS and cutting into 1–3 mm^3^ pieces. Filter the impurity through strainer and discard the supernatant after centrifugation. After mixing the cell pellet with matrix gel, cells were plant into 24 well culture plate and cultured in incubator (37 °C, 5% CO_2_).

### Tumor xenografts

Female BALB/c nude mice (four weeks of age) were purchased and housed at the Laboratory Animal Science of Fudan University Shanghai Cancer Center. One week after adaptive feeding, 2 × 106 A2780 cells stably expressing shNC or shUSP43 were injected subcutaneously into the right armpit region of each BALB/c nude mouse. The mice and tumor volumes were weighed every 3–5 d. Tumor volume was calculated using the following formula: volume = length × width^2^ × 0.5. Once the tumor volume reached 1500 mm^3^, the mice were euthanized to harvest the tumors.

For the cisplatin and SLC7A11 inhibitor combination assay, A2780 cells stably overexpressing USP43 and A2780 control cells were injected into nude mice. Mice injected with USP43-overexpressing cells were randomly divided into four groups and treated with PBS, cisplatin alone (4 mg/kg), HG106 alone (3 mg/kg), or cisplatin in combination with HG106. HG106 received daily intraperitoneal administration, whereas cisplatin was administered every 3–4 days via intraperitoneal injection. The mice were weighed and sacrificed as described above.

### Statistical analysis

All statistical analyses were performed using R software (4.2.2). All experimental results are presented as the mean ± standard error (SEM) from three independent biological replicates.

Statistical differences were assessed using two-tailed unpaired Student’s *t*-test, Wilcoxon test, or chi-square (*χ*^2^) test for comparisons between each group. Statistical significance between multiple groups was evaluated using one-way or two-way analysis of variance (ANOVA). The Kaplan–Meier method was used to compare significant differences between patient groups. Spearman’s rank correlation coefficient was used to determine the strength of expression correlations between different genes. *p* values less than 0.05 were considered statistically significant (*p < 0.05, **p < 0.01, and ***p < 0.001).

## Supplementary information

USP43 expression across normal and tumor tissues

USP43 influences apoptosis of ovarian cancer cells

USP43 influences the SLC7A11 expression without combination to it

FASN could rescue the ferroptosis-related phenomenon changed by USP43

The DDP resistance and protein expression levels of A2780-DDP and SKOV3-DDP cell lines

Induction of SLC7A11 can restore the inhibitory effects of USP43-ablation on the malignant phenotype of OC in DDP-resistant cell lines

The body weight change curves of nude mice

Western blot assay shows the knockdown and overexpression efficiency of YY1

IP-MASS results in USP43-overexpression A2780 cells

Primer sequences

full uncropped Gels and Blots image(s)

## Data Availability

Research data can be obtained from public databases.
