# Supplementary figures and images for: YY1-induced USP43 drives ferroptosis suppression by FASN stabilization and subsequent activation of SLC7A11 in ovarian cancer
Source: Cell Death Dis. 2025 Sep 1;16(1):589. doi: 10.1038/s41419-025-07886-5 (PMC12402158; doi:10.1038/s41419-025-07886-5)

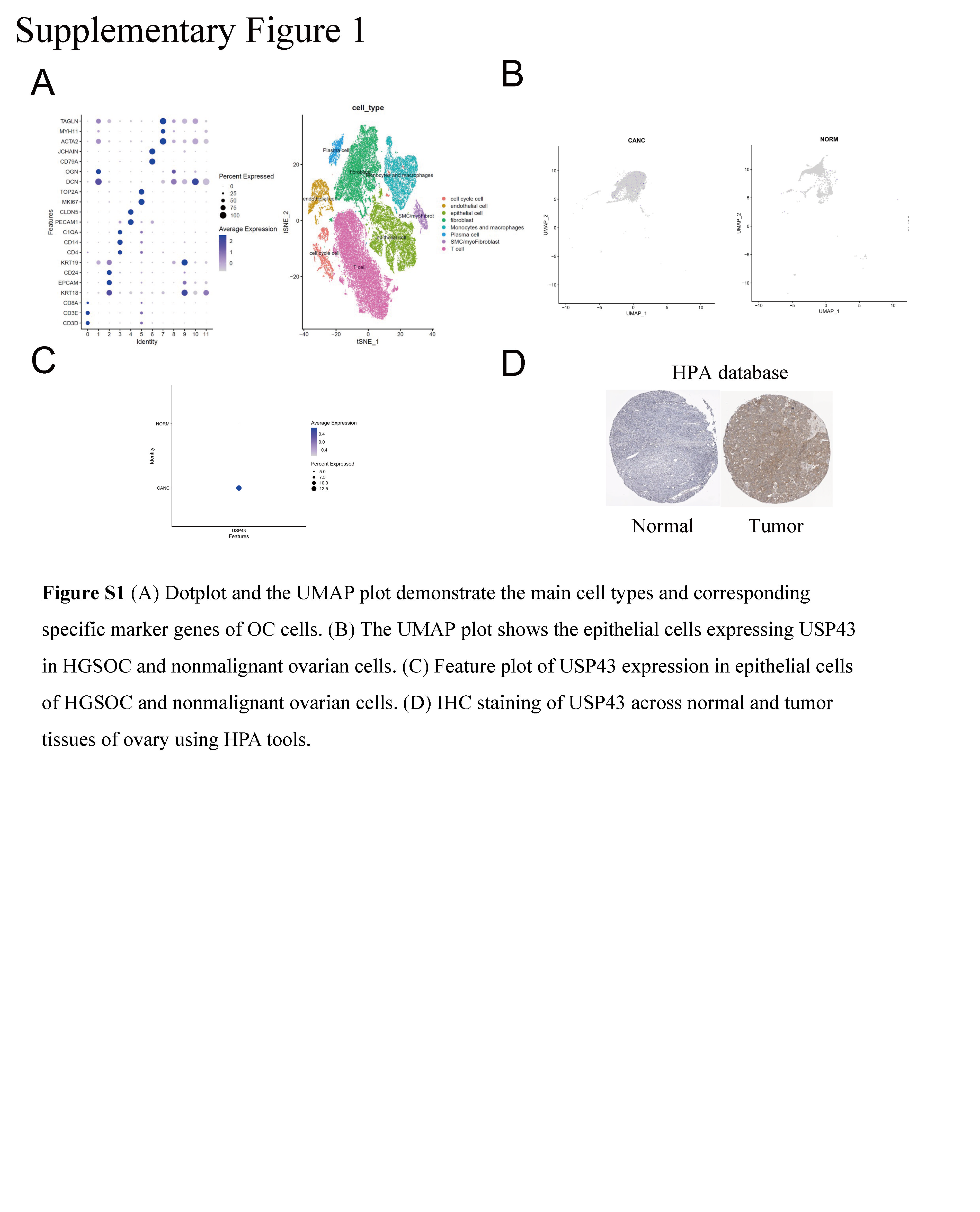

Supplement: Supplementary file 1 — USP43 expression across normal and tumor tissues [file 41419_2025_7886_MOESM1_ESM.tif]

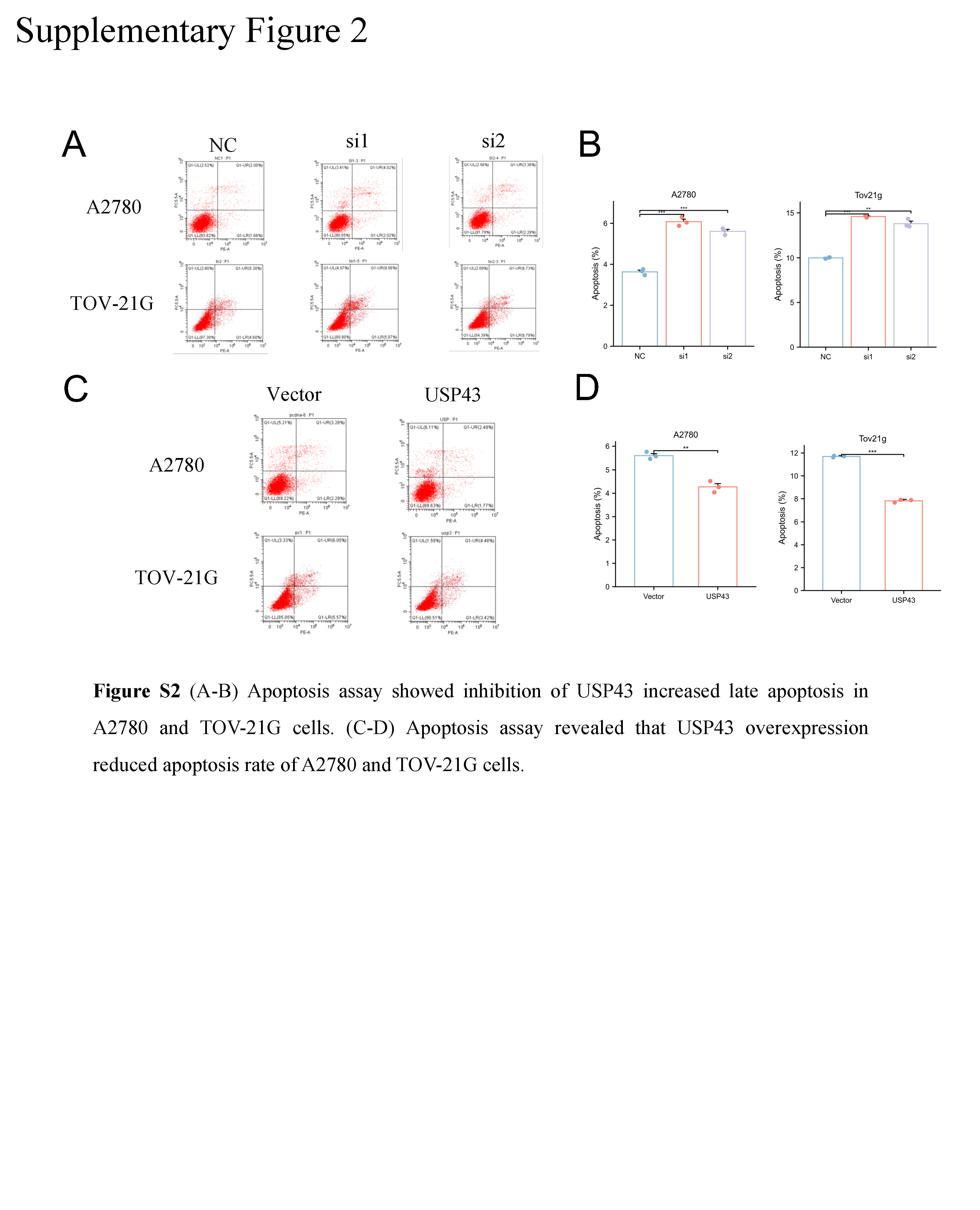

Supplement: Supplementary file 2 — USP43 influences apoptosis of ovarian cancer cells [file 41419_2025_7886_MOESM2_ESM.tif]

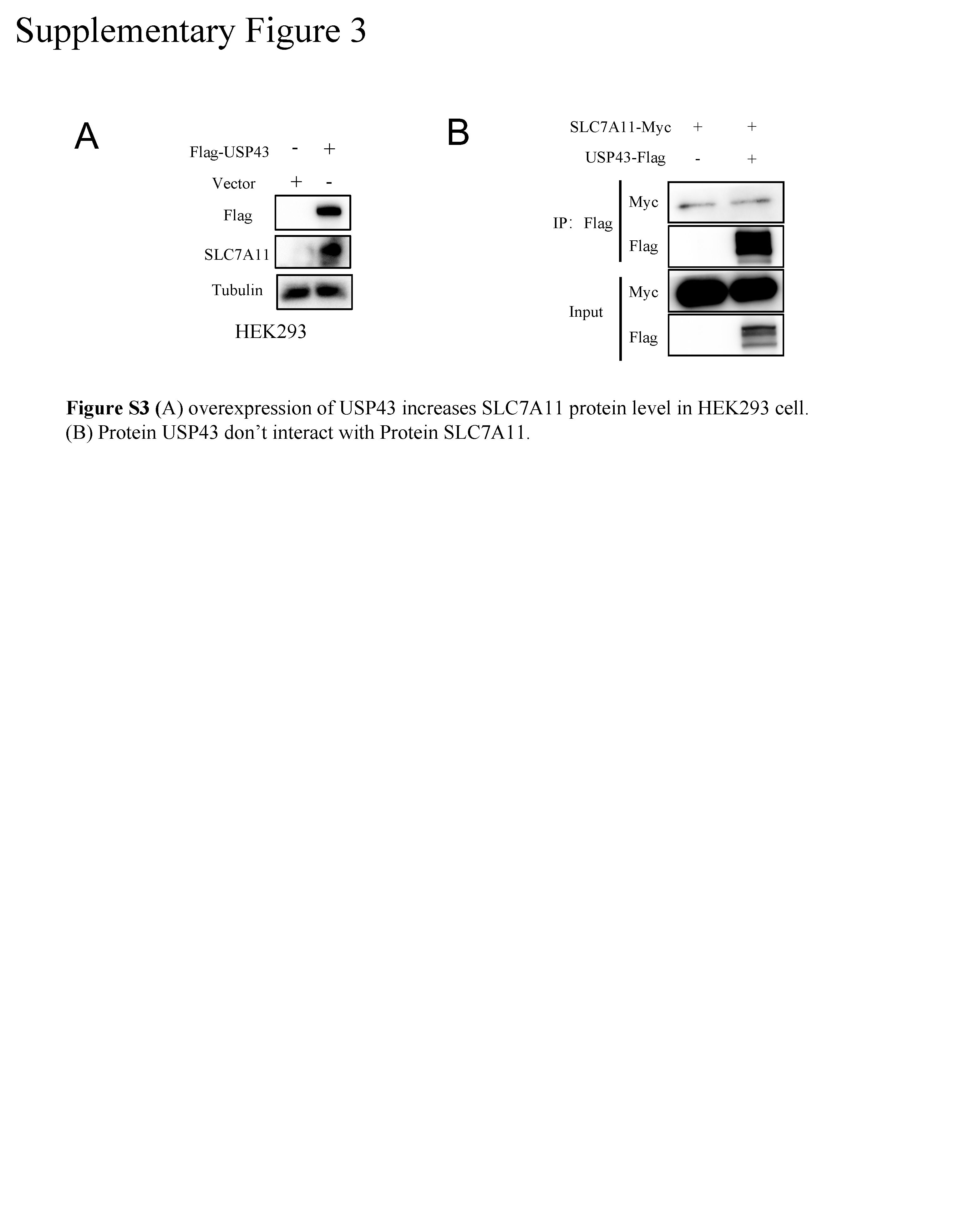

Supplement: Supplementary file 3 — USP43 influences the SLC7A11 expression without combination to it [file 41419_2025_7886_MOESM3_ESM.tif]

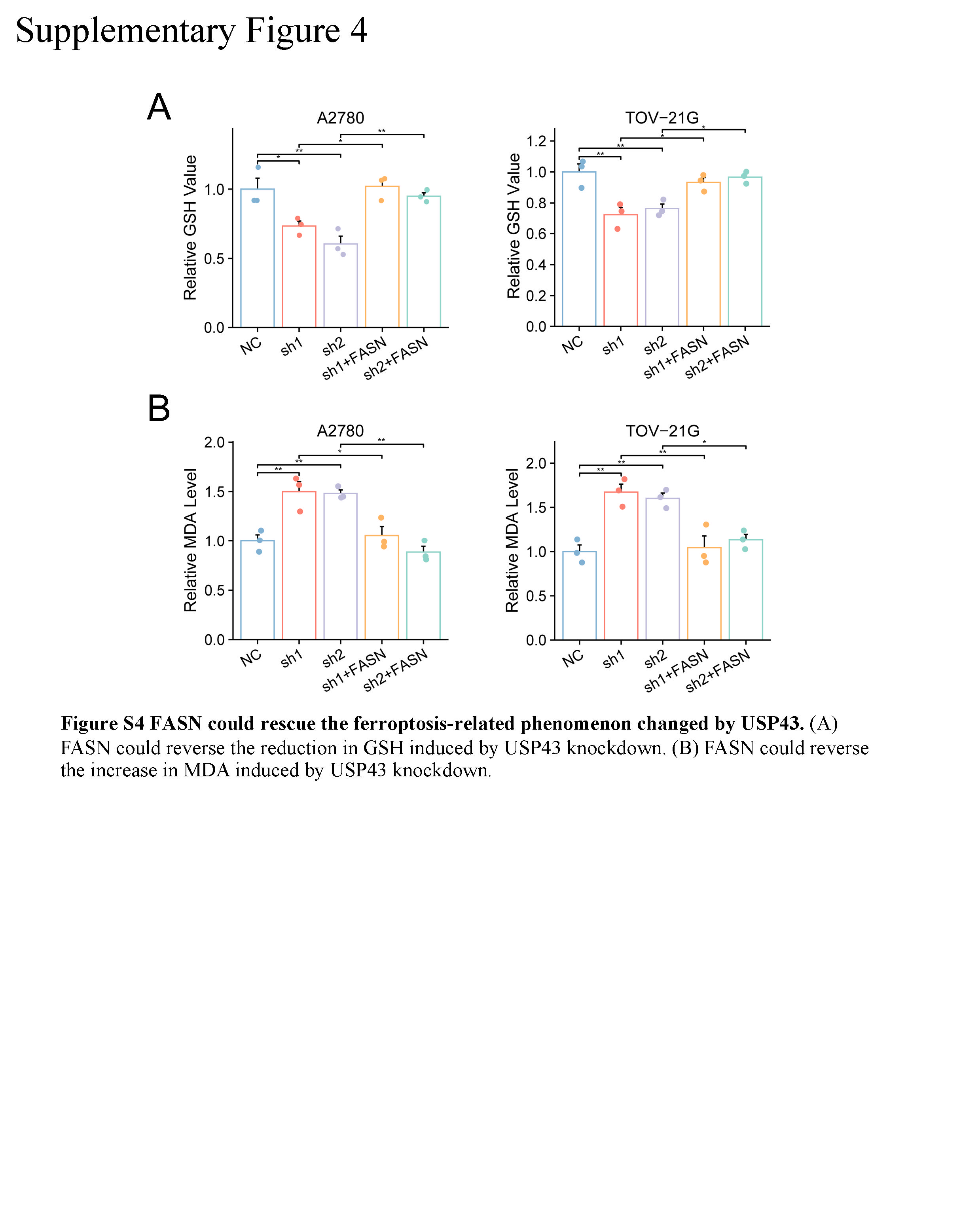

Supplement: Supplementary file 4 — FASN could rescue the ferroptosis-related phenomenon changed by USP43 [file 41419_2025_7886_MOESM4_ESM.tif]

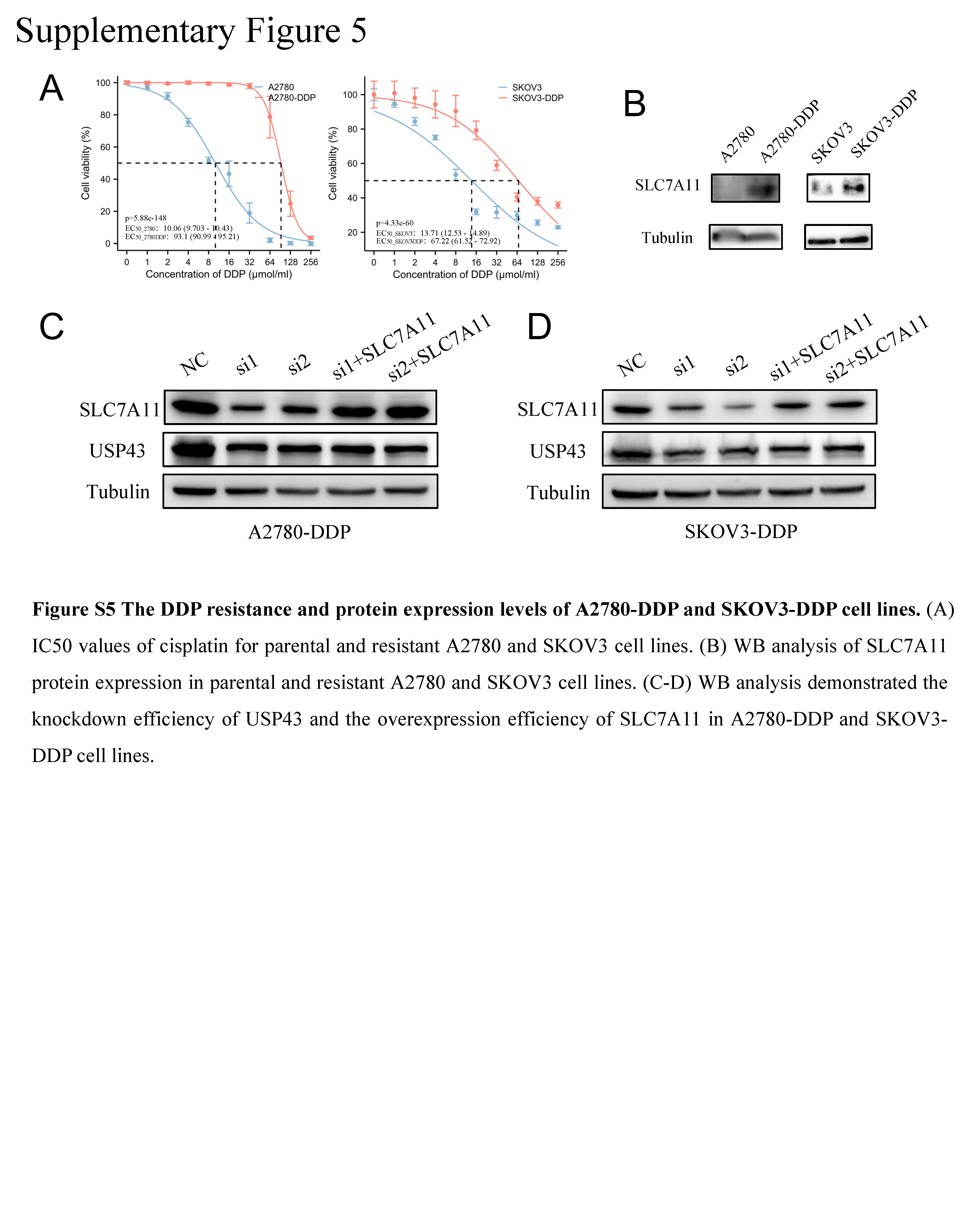

Supplement: Supplementary file 5 — The DDP resistance and protein expression levels of A2780-DDP and SKOV3-DDP cell lines [file 41419_2025_7886_MOESM5_ESM.tif]

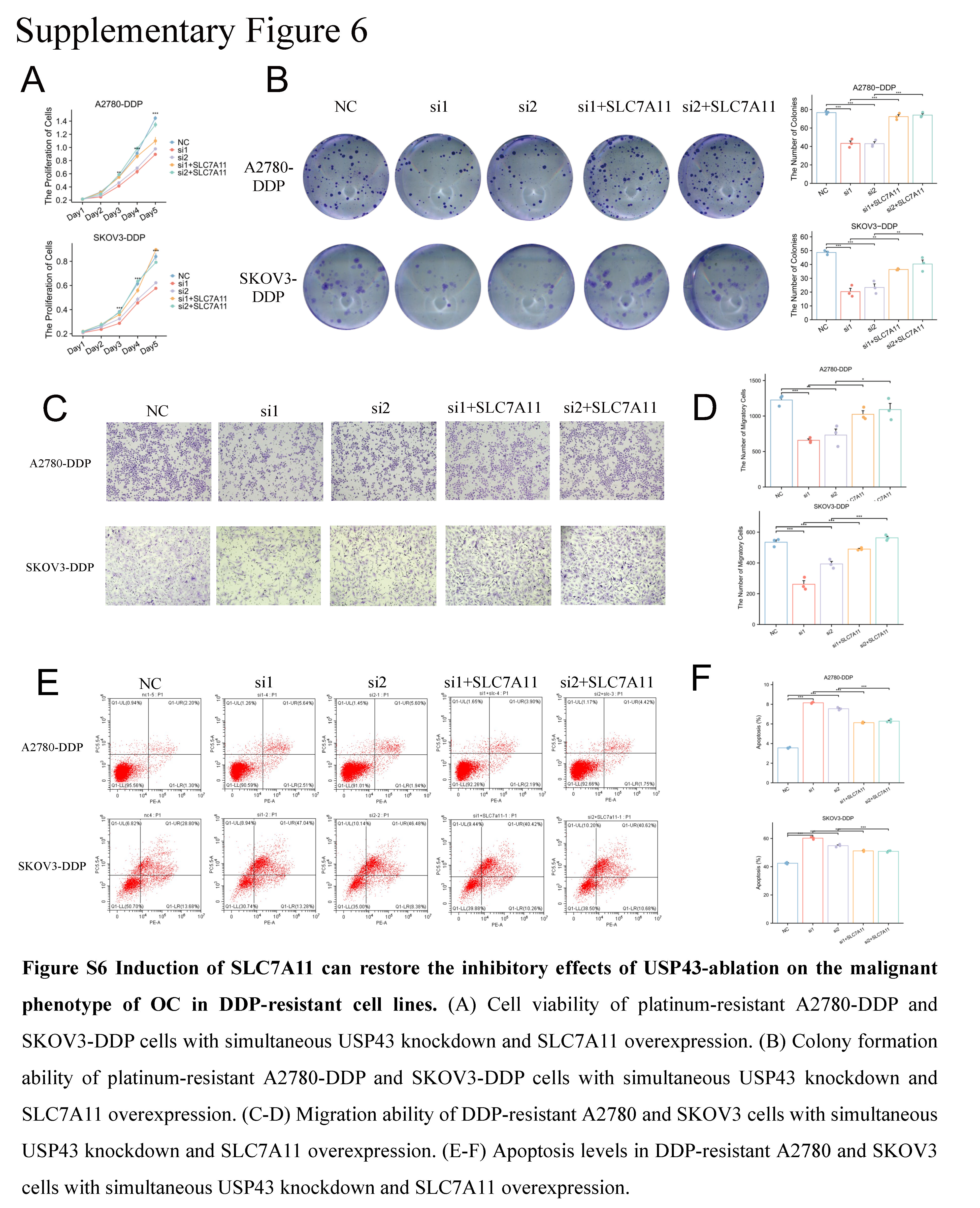

Supplement: Supplementary file 6 — Induction of SLC7A11 can restore the inhibitory effects of USP43-ablation on the malignant phenotype of OC in DDP-resistant cell lines [file 41419_2025_7886_MOESM6_ESM.tif]

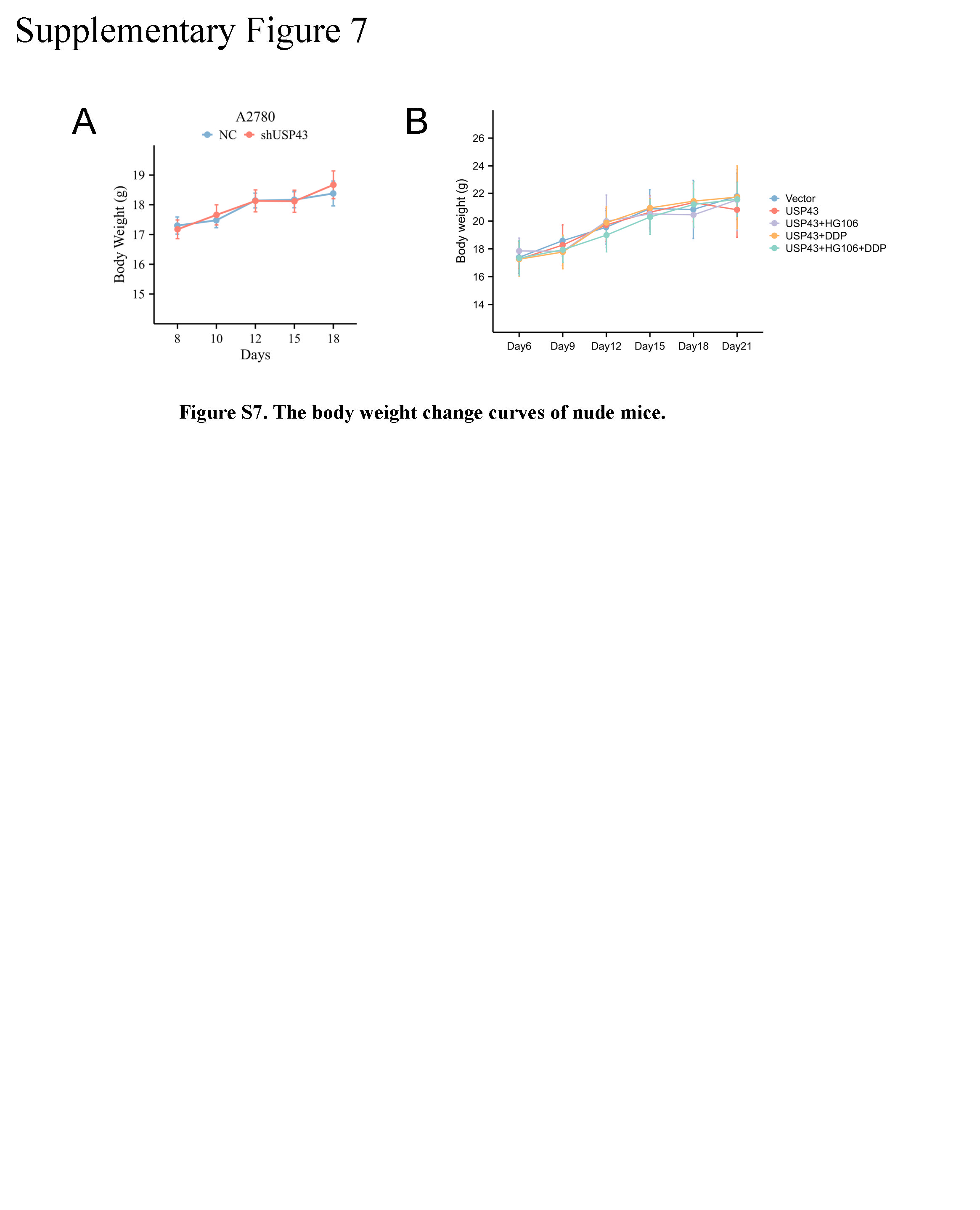

Supplement: Supplementary file 7 — The body weight change curves of nude mice [file 41419_2025_7886_MOESM7_ESM.tif]

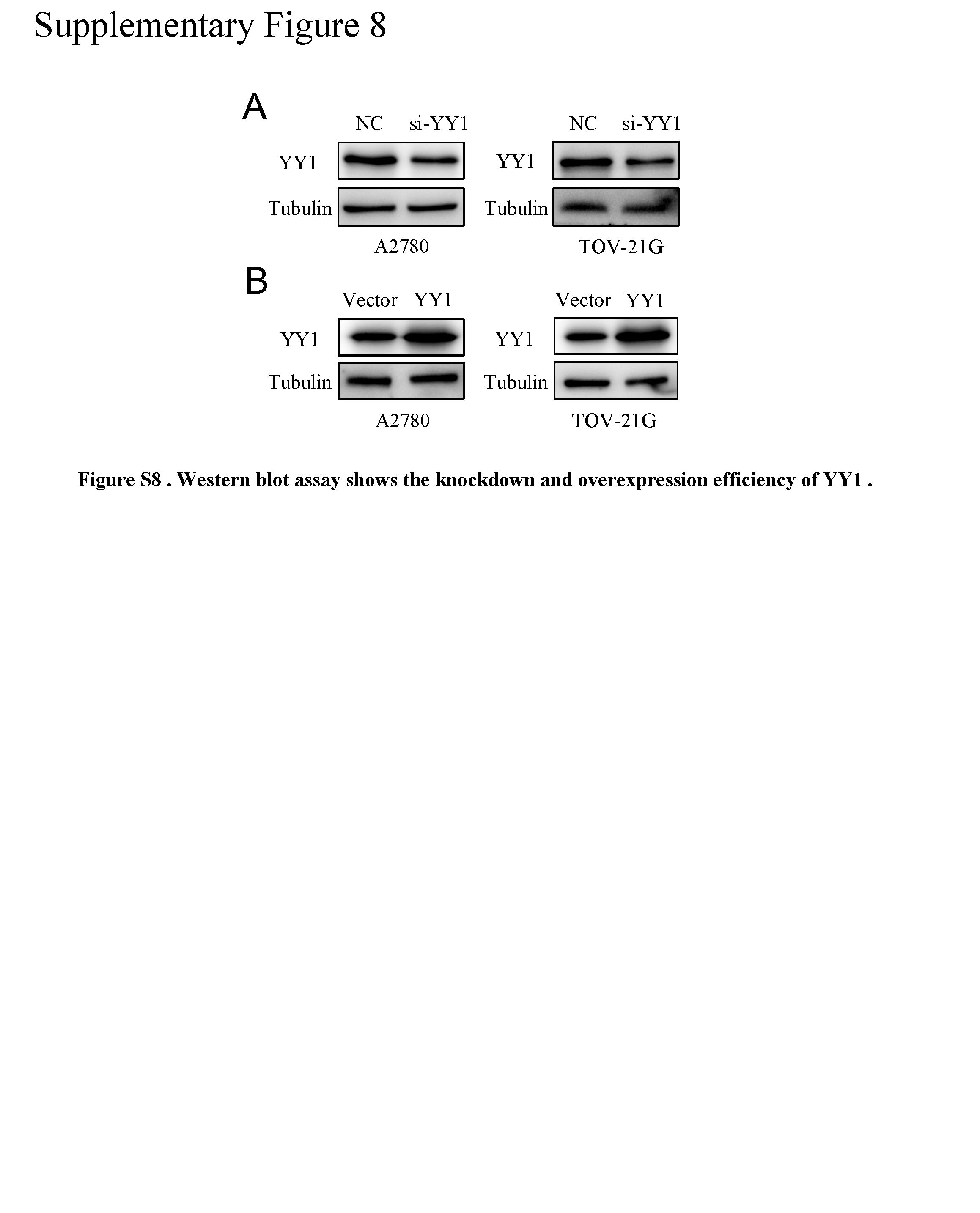

Supplement: Supplementary file 8 — Western blot assay shows the knockdown and overexpression efficiency of YY1 [file 41419_2025_7886_MOESM8_ESM.tif]
