# Supplementary material for: YY1-induced USP43 drives ferroptosis suppression by FASN stabilization and subsequent activation of SLC7A11 in ovarian cancer
Source: Cell Death Dis. 2025 Sep 1;16(1):589. doi: 10.1038/s41419-025-07886-5 (PMC12402158; doi:10.1038/s41419-025-07886-5)

Figure 2A-21g-tubulin

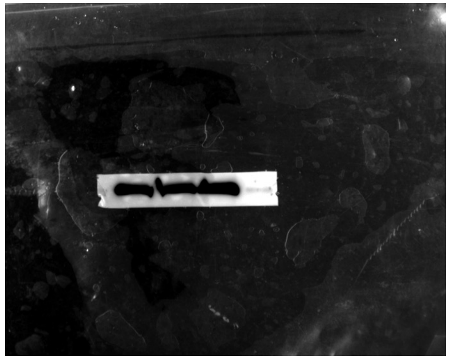

Figure 2A-21g-usp43

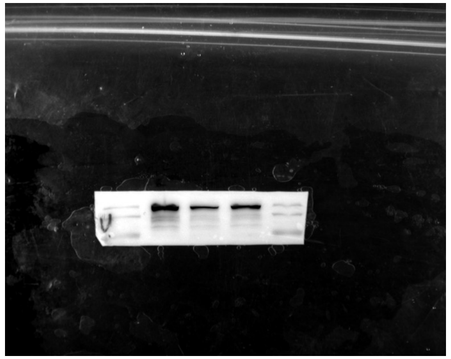

Figure 2A-27-tubulin

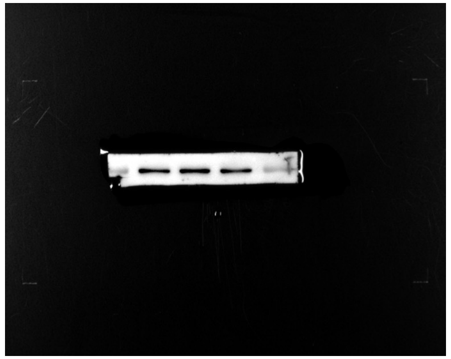

Figure 2A-27-usp43

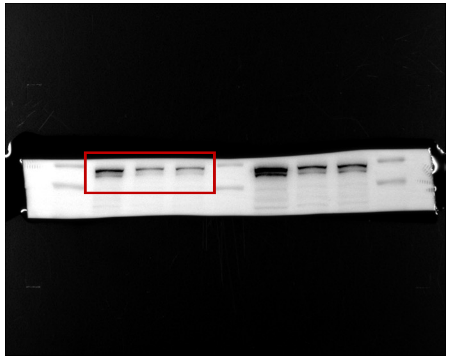

Figure 2E-21g-tubulin

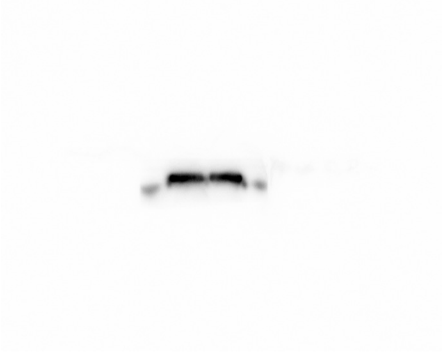

Figure 2E-21g-usp43

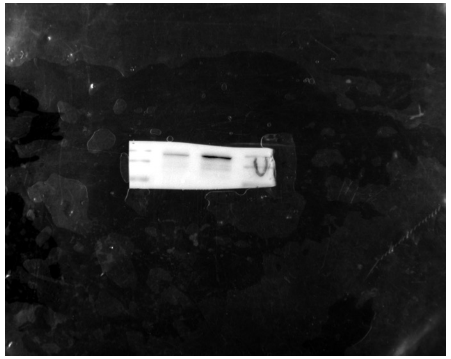

Figure 2E-27-tubulin

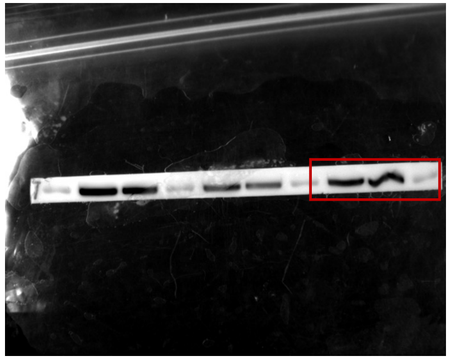

Figure 2E-27-usp43

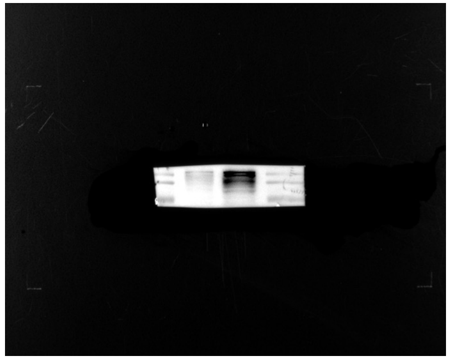

Figure 2I-21g-E-cadherin

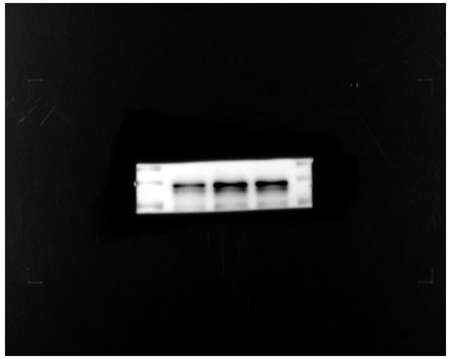

Figure 2I-21g-n-cadherin

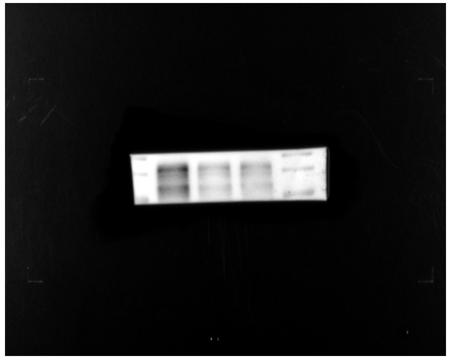

Figure 2I-21g-tubulin

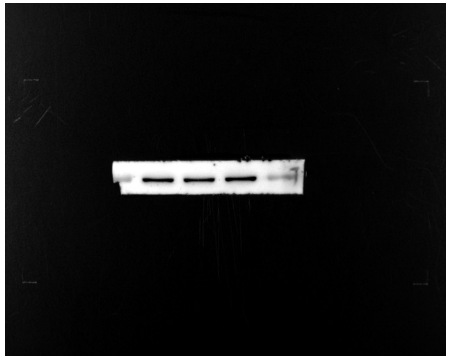

Figure 2I-21g-vimentin

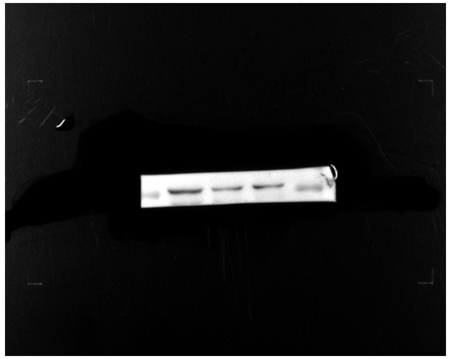

Figure 2I-27-E-cadherin

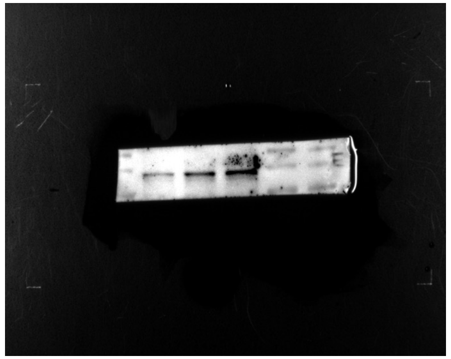

Figure 2I-27-n-cadherin

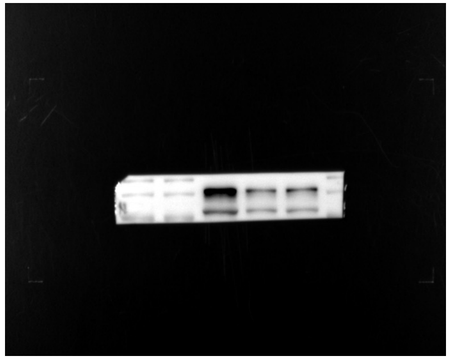

Figure 2I-27-tubulin

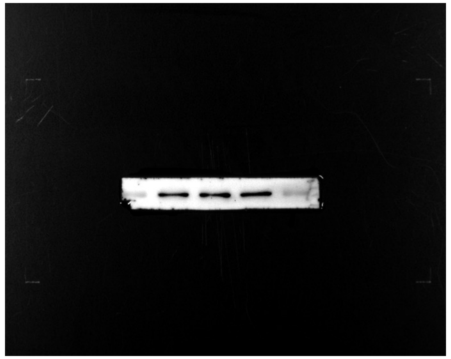

Figure 2I-27-vimentin

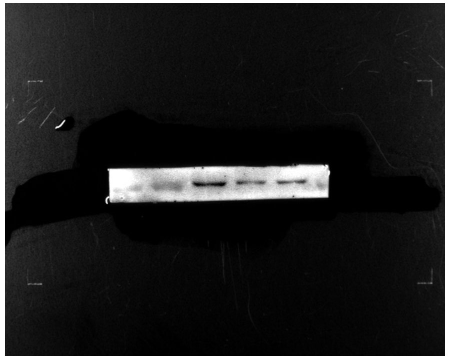

Figure 2J-21g-E-cadherin

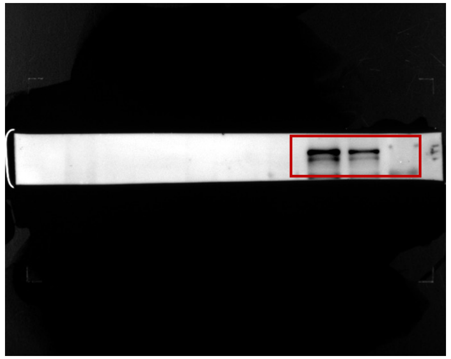

Figure 2J-21g-n-cadherin

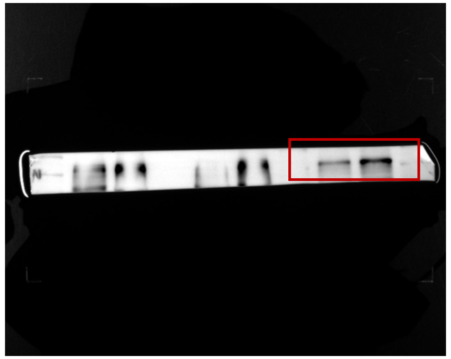

Figure 2J-21g-tubulin

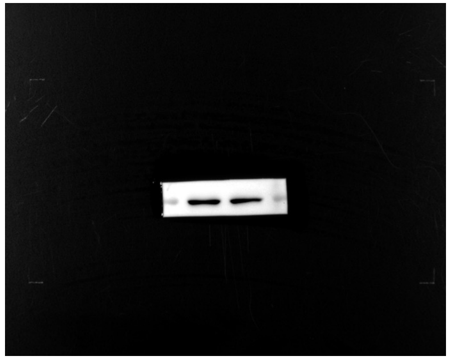

Figure 2J-21g-vimentin

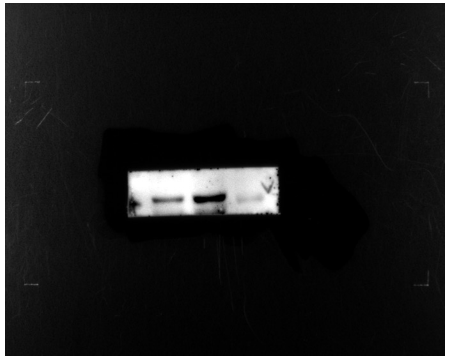

Figure 2J-27-E-cadherin

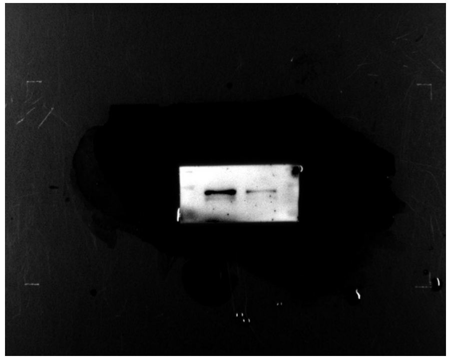

Figure 2J-27-n-cadherin

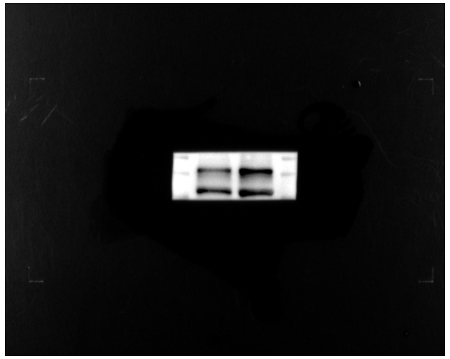

Figure 2J-27-tubulin

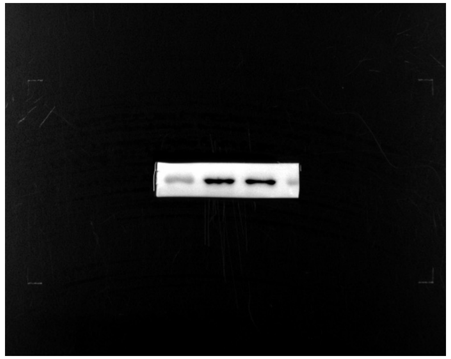

Figure 2J-27-vimentin

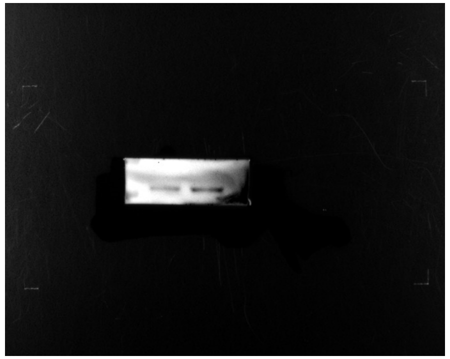

Figure 4E-21g-SLC7A11

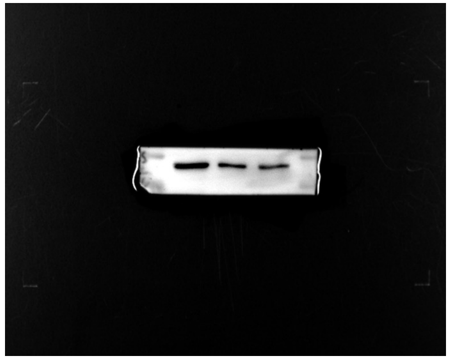

Figure 4E-21g-tubulin

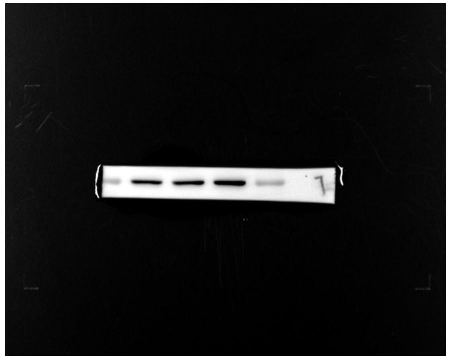

Figure 4E-21g-usp43

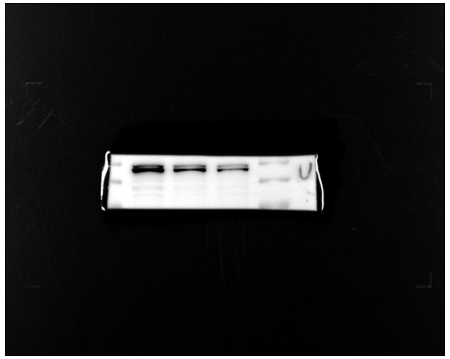

Figure 4E-27-SLC7A11

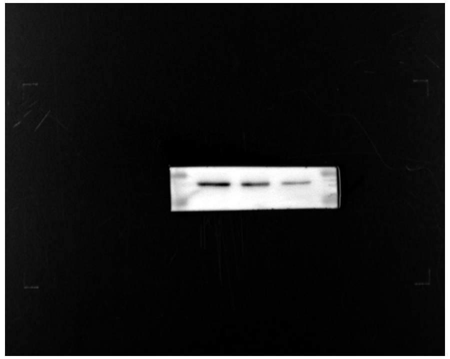

Figure 4E-27-tubulin

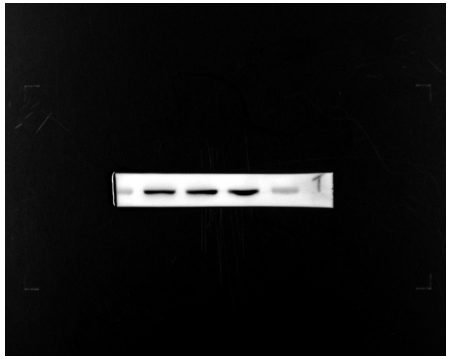

Figure 4E-27-USP43

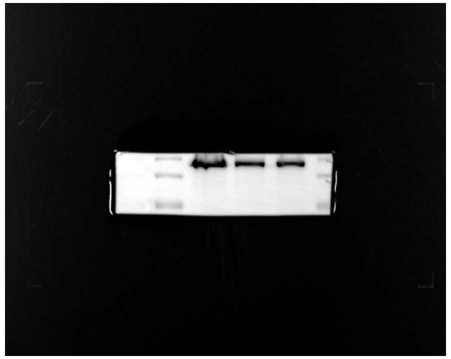

Figure 4E-R3-SLC7A11

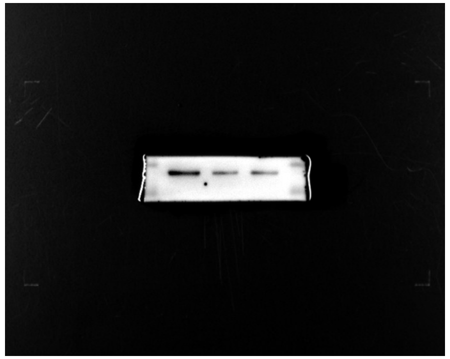

Figure 4E-R3-tubulin

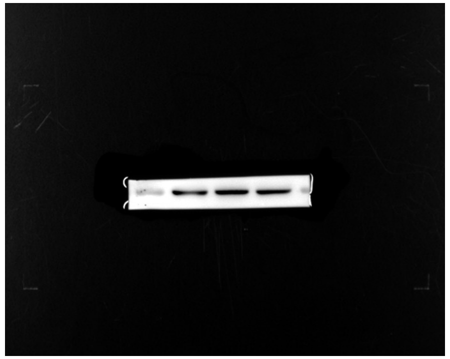

Figure 4E-R3-USP43

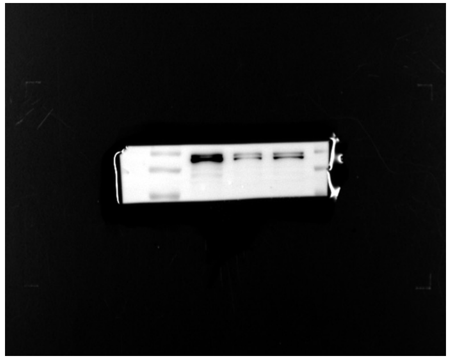

Figure 4E-SK-SLC7A11

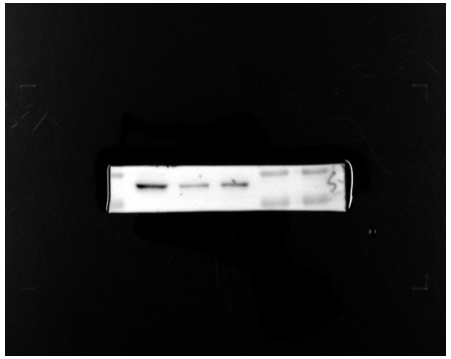

Figure 4E-SK-tubulin

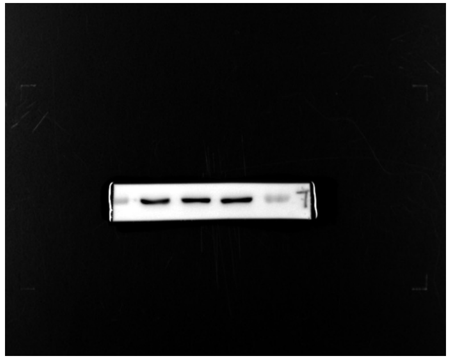

Figure 4E-SK-USP43

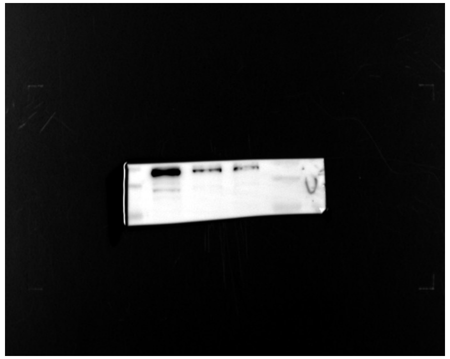

Figure 4F-21g-flag

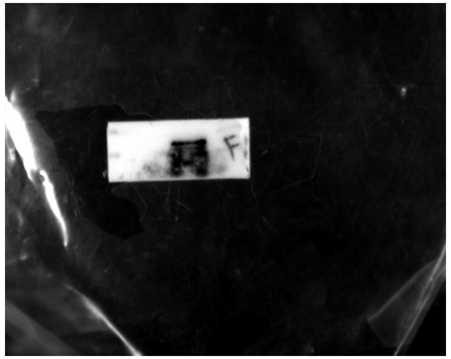

Figure 4F-21g-SLC7A11

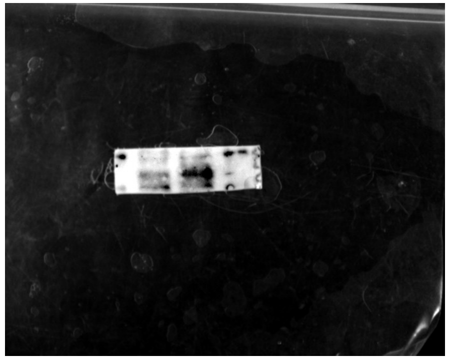

Figure 4F-21g-tubulin

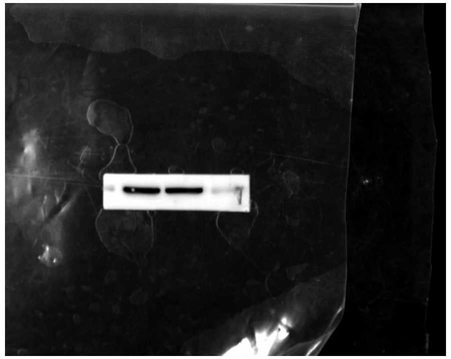

Figure 4F-27-flag

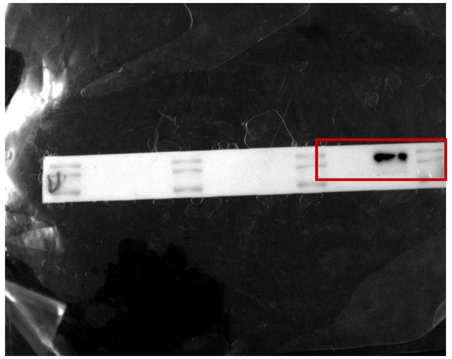

Figure 4F-27-SLC7A11

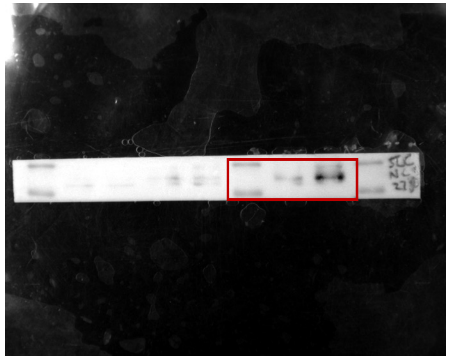

Figure 4F-27-tubulin

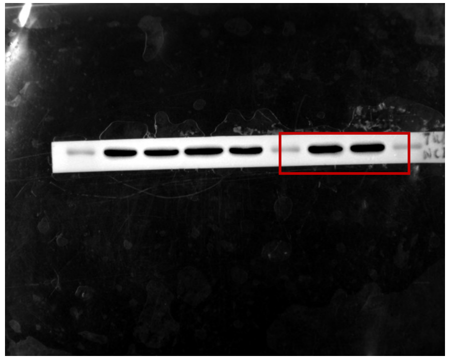

Figure 4F-R3-flag

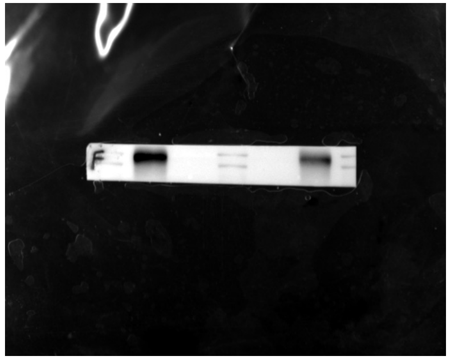

Figure 4F-R3-SLC7A11

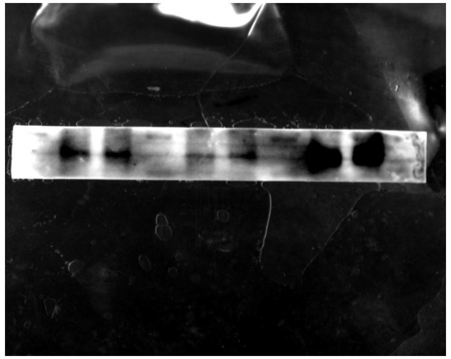

Figure 4F-R3-tubulin

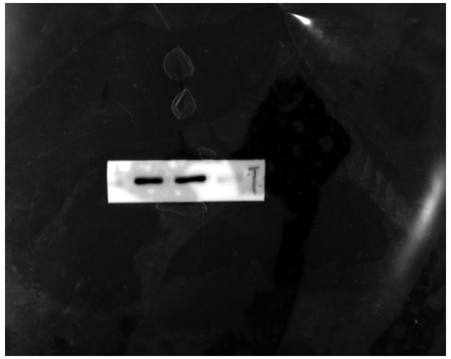

Figure 4F-SK-Flag

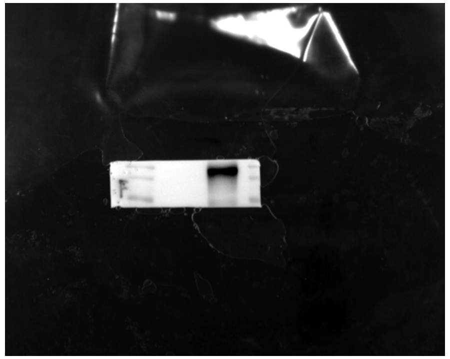

Figure 4F-SK-SLC7A11

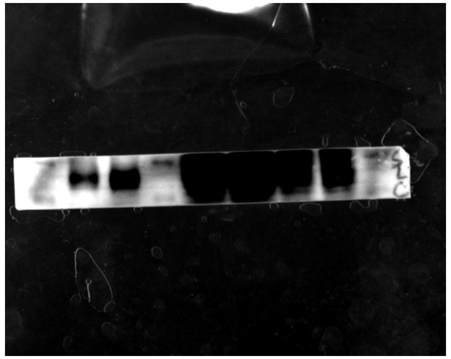

Figure 4F-SK-tubulin

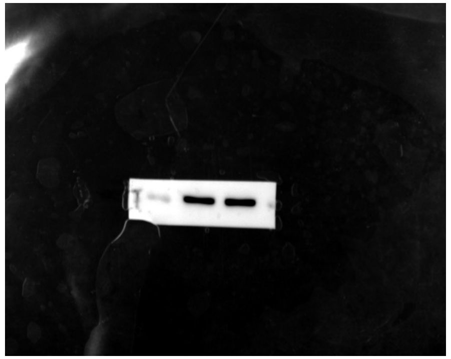

Figure 5A-Input-flag

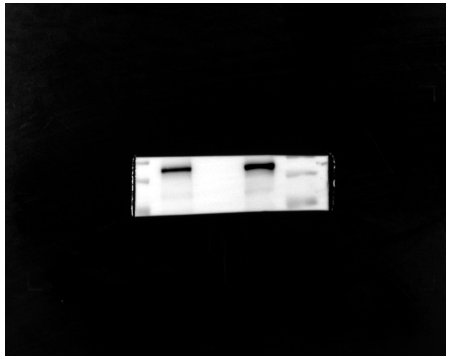

Figure 5A-input-myc

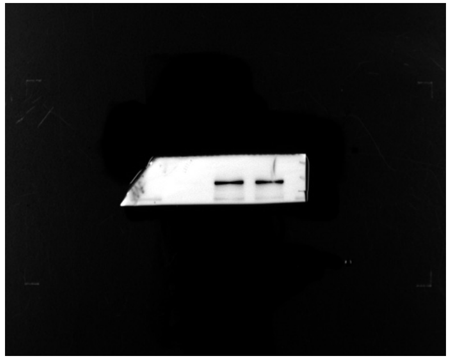

Figure 5A-IP-Flag

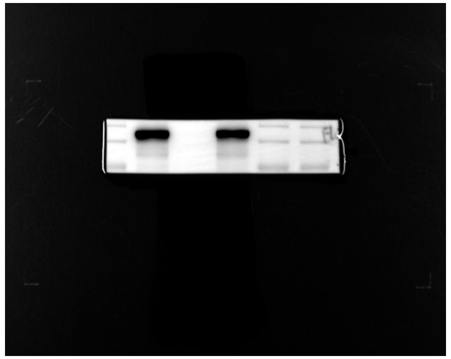

Figure 5A-IP-myc

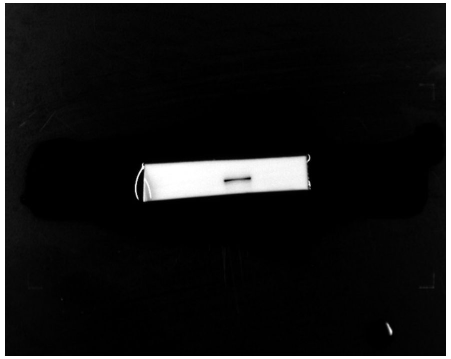

Figure 5B-input-flag

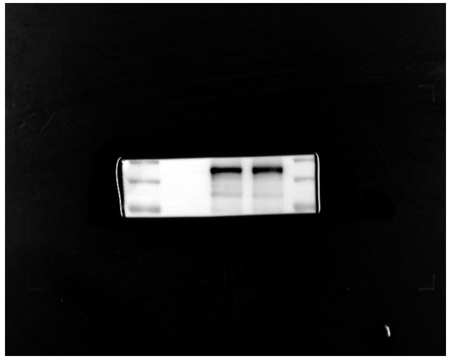

Figure 5B-input-myc

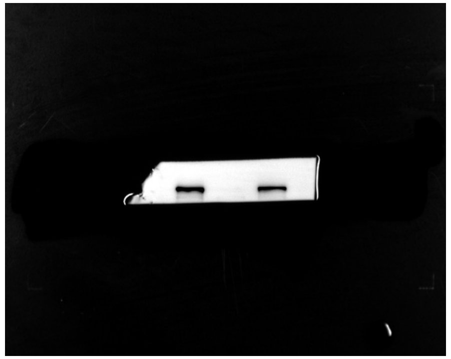

Figure 5B-IP-flag

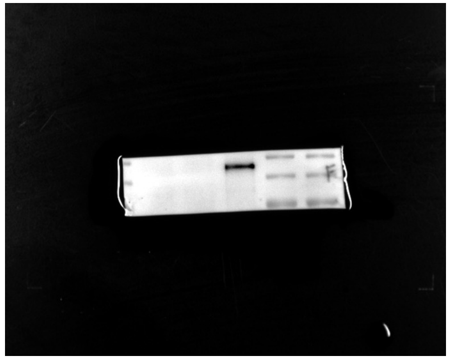

Figure 5B-IP-myc

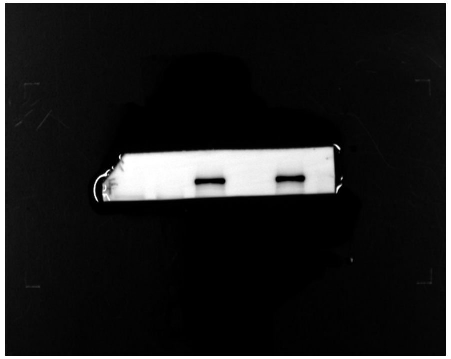

Figure 5C-21g-ip-FASN

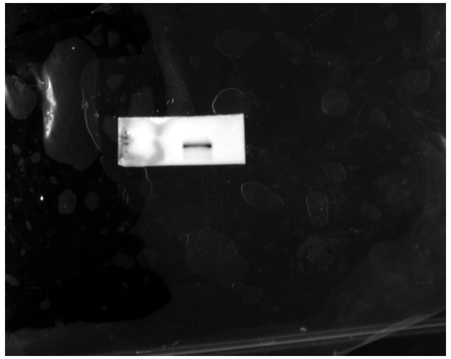

Figure 5C-21g-ip-IgG

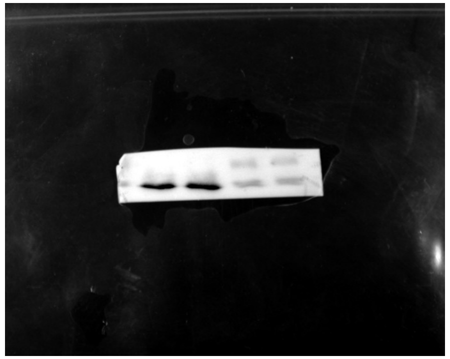

Figure 5C-21g-ip-USP43

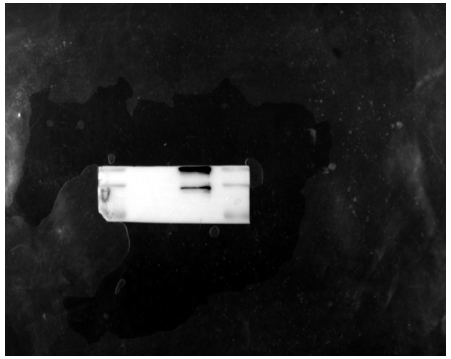

Figure 5C-27-input-FASN

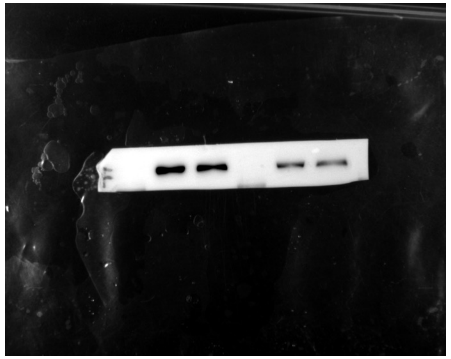

Figure 5C-27-input-USP43

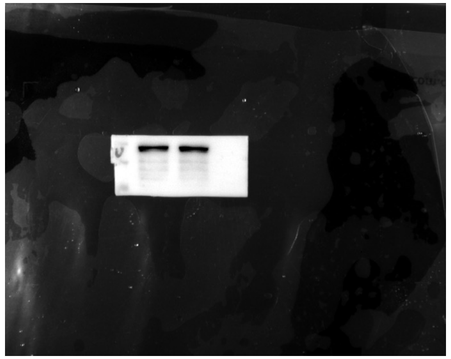

Figure 5C-27-ip-FASN

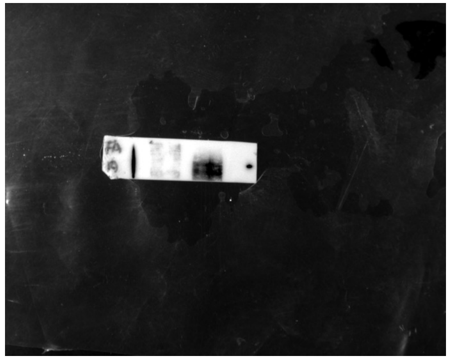

Figure 5C-27-ip-IgG

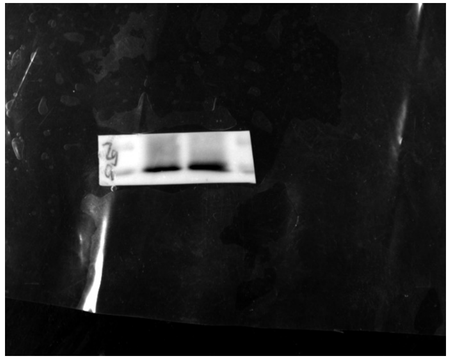

Figure 5C-27-ip-USP43

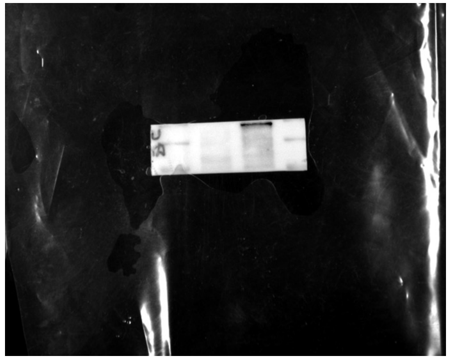

Figure 5E-21g-FASN

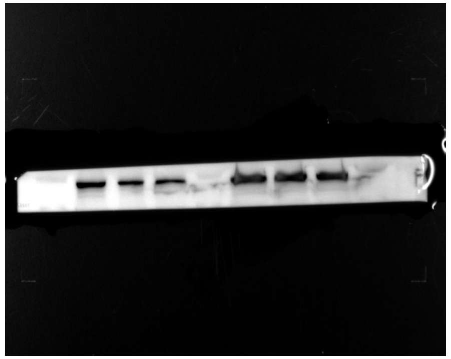

Figure 5E-21g-tubulin

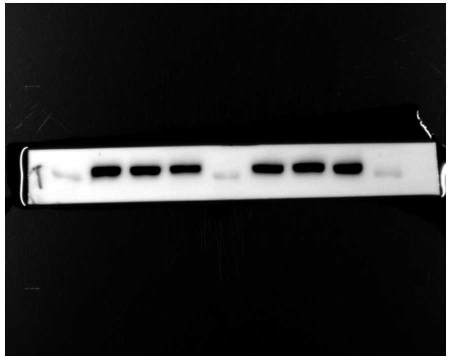

Figure 5E-21g-USP43

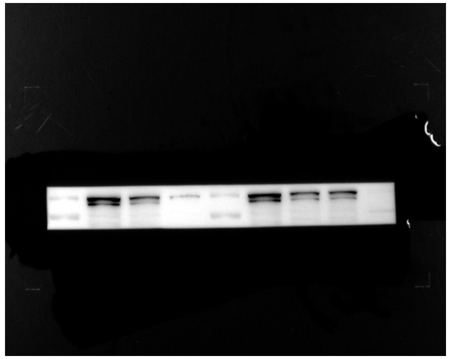

Figure 5E-27-FASN

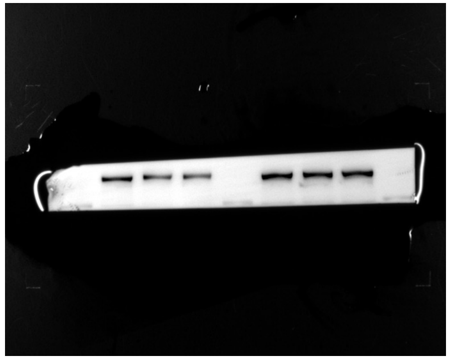

Figure 5E-27-tubulin

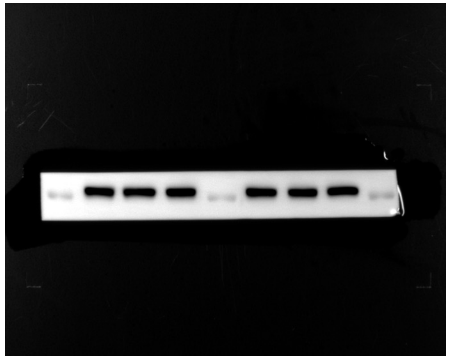

Figure 5E-27-USP43

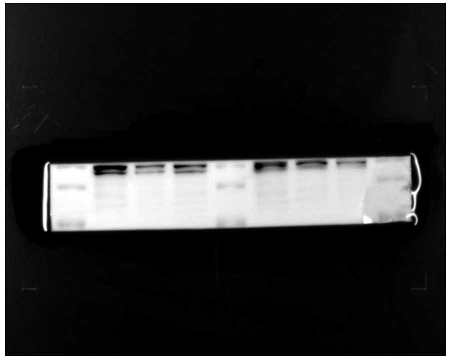

Figure 5F-21g-FASN

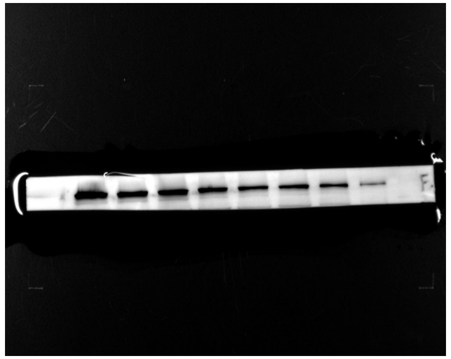

Figure 5F-21g-tubulin

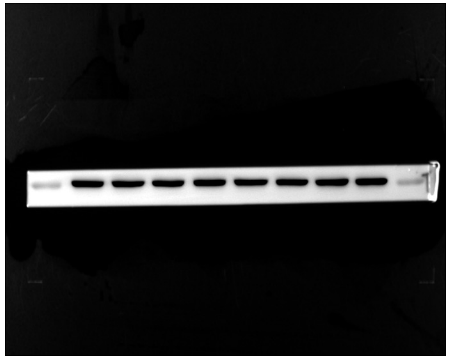

Figure 5F-27-FASN

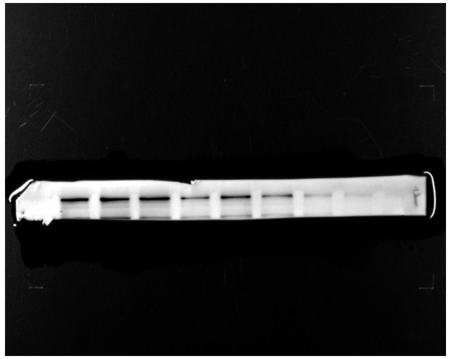

Figure 5F-27-tubulin

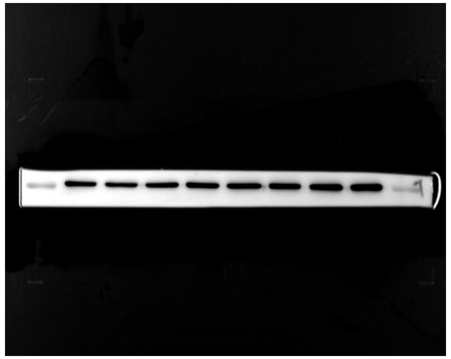

Figure 5G-FASN-right

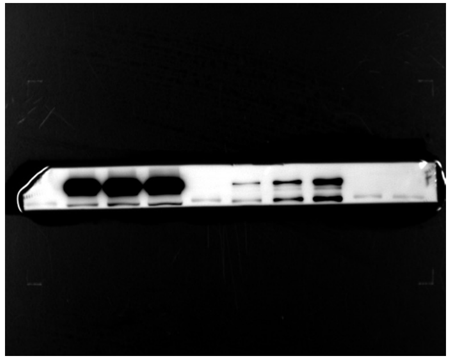

Figure 5G-tubulin-left

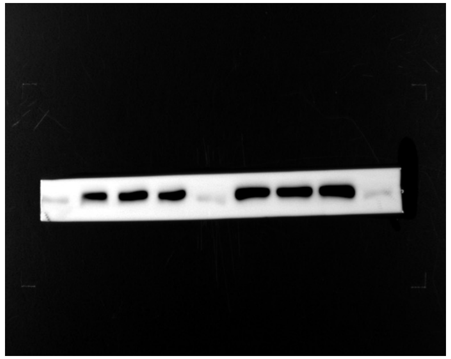

Figure 5G-USP43-left

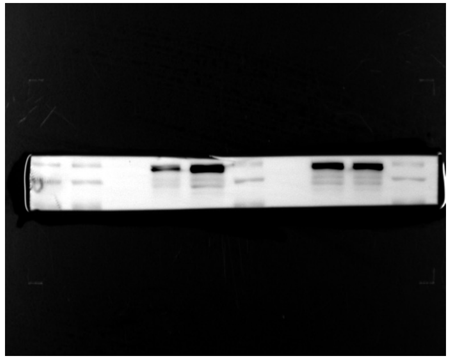

Figure 5H-FASN

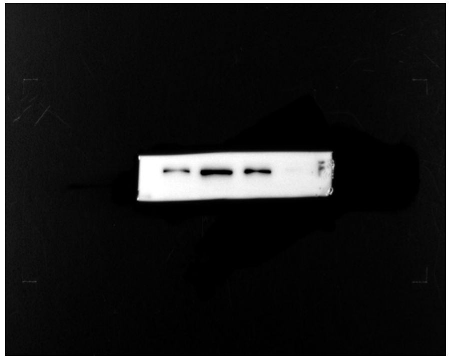

Figure 5H-USP43-right

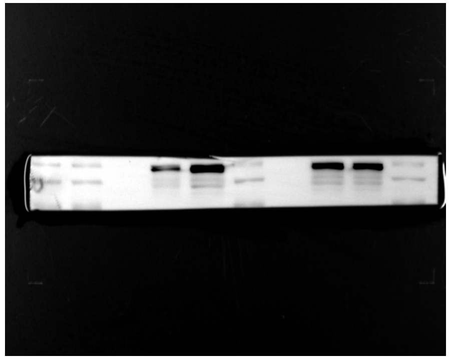

Figure 5H-tubulin-right

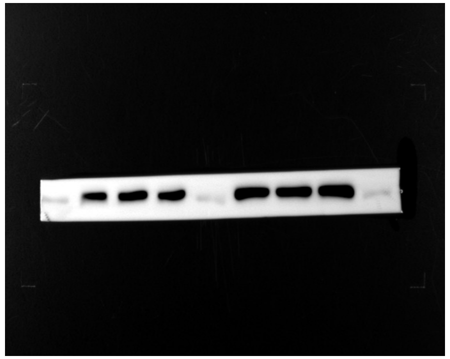

Figure 6A-27-tubulin

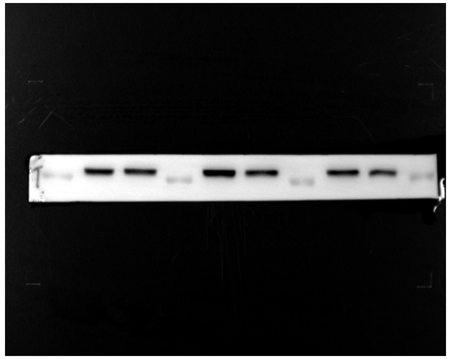

Figure 6A-27-ub

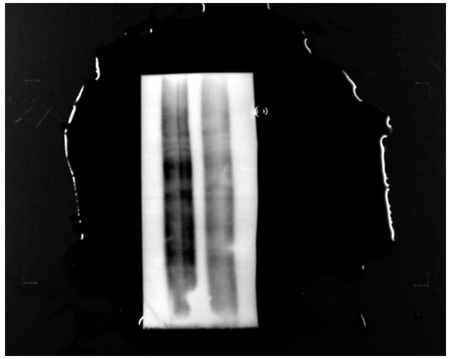

Figure 6A-27-USP43

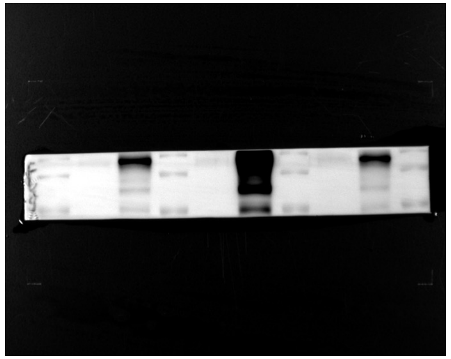

Figure 6A-21g-FASN

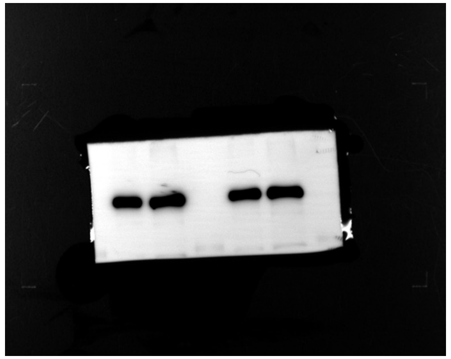

Figure 6A -21g-tubulin

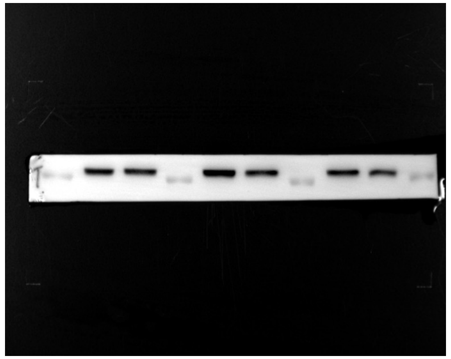

Figure 6A -21g-ub

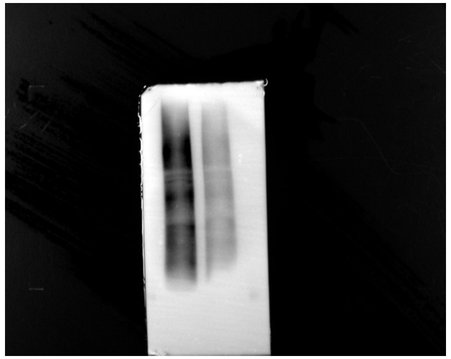

Figure 6A -21g-USP43

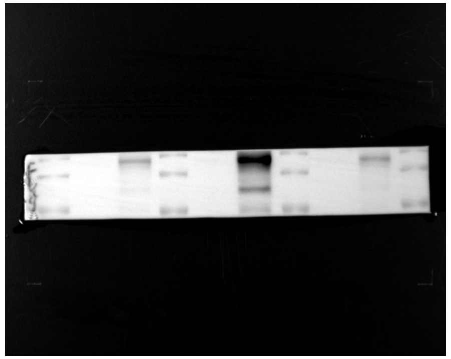

Figure 6A-27-FASN

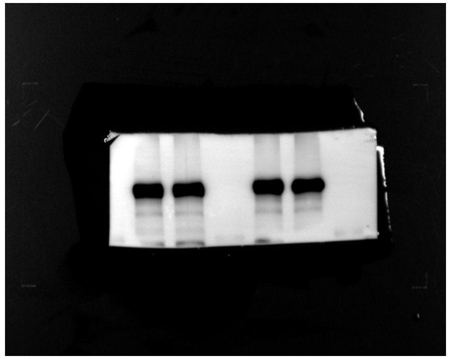

Figure 6D-Input-Myc

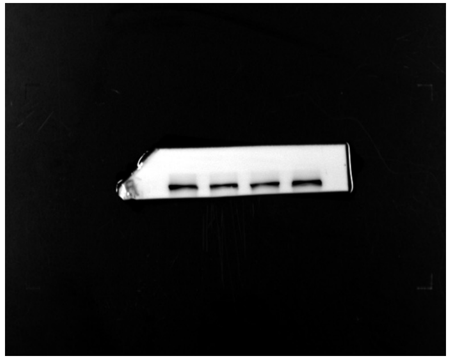

Figure 6D-Input-Flag

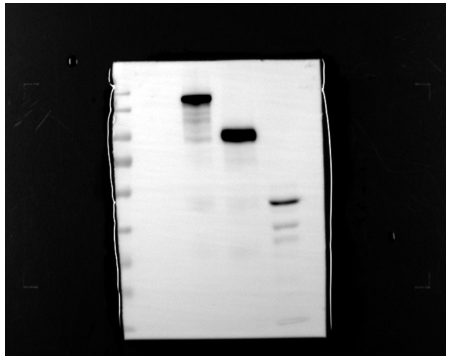

Figure 6D-IP-Flag

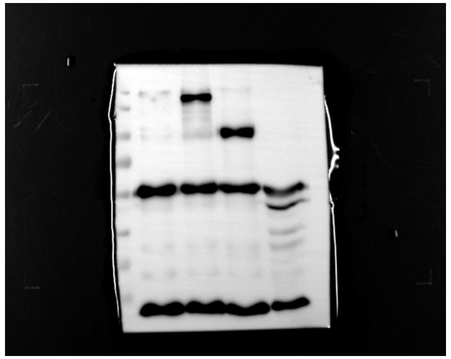

Figure 6D-IP-Myc

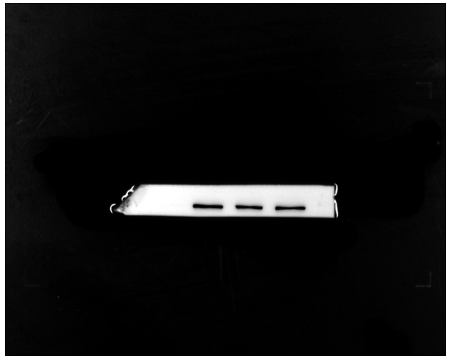

Figure 6B-IP-HA

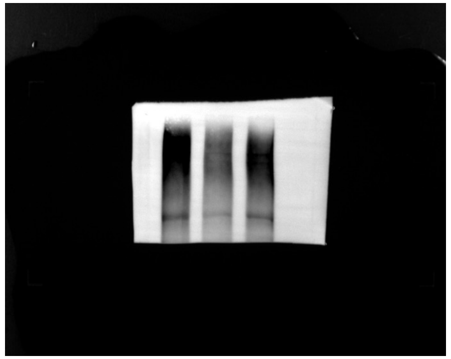

Figure 6B-IP-Myc

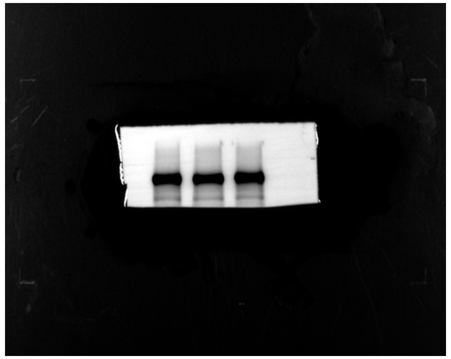

Figure 6B-Input-Myc

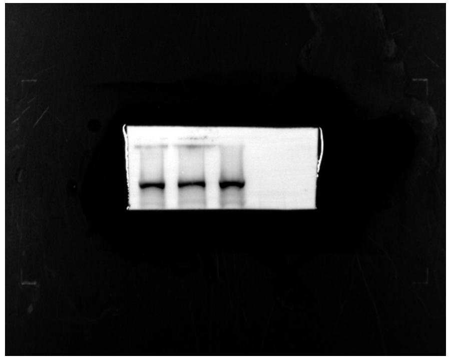

Figure 6B-Input-Flag

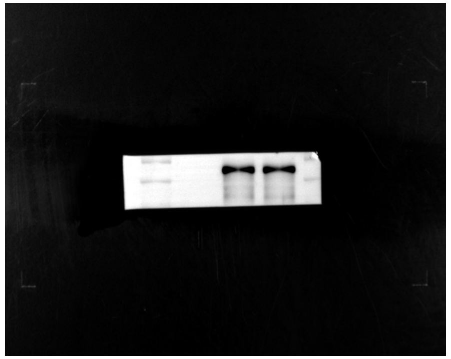

Figure 6E-input-flag

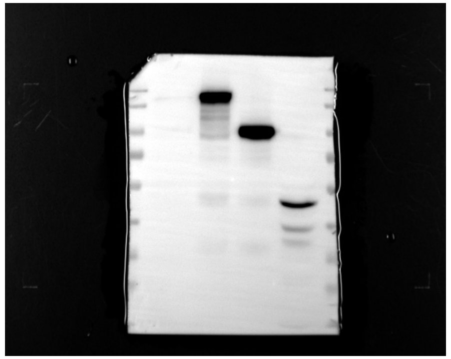

Figure 6E-input-myc

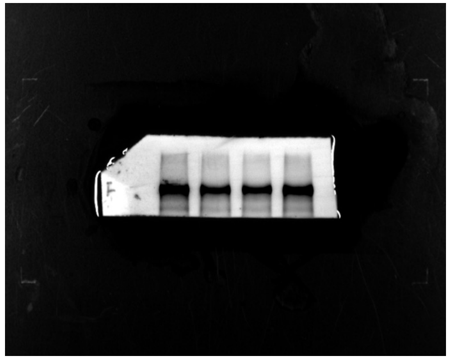

Figure 6E-IP-myc

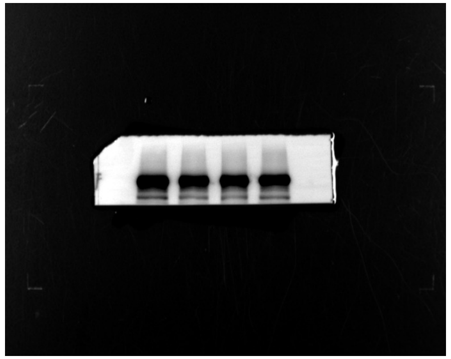

Figure 6E-IP-HA

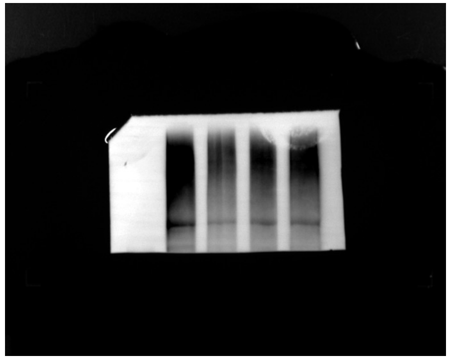

Figure 7A-21g-FASN

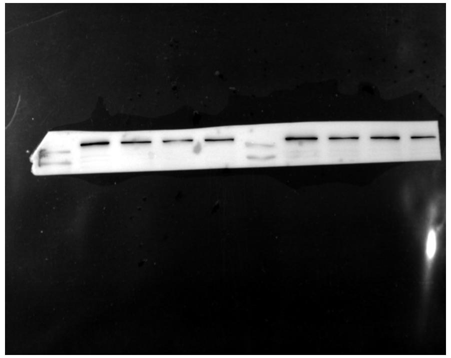

Figure 7A-21g-HIF1a

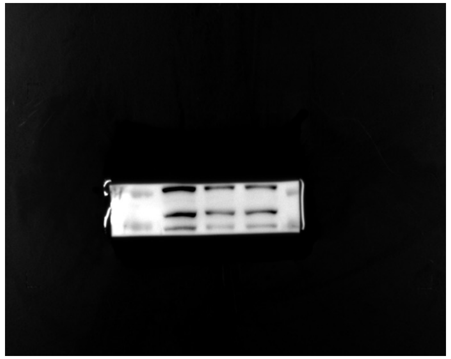

Figure 7A-21g-tubulin

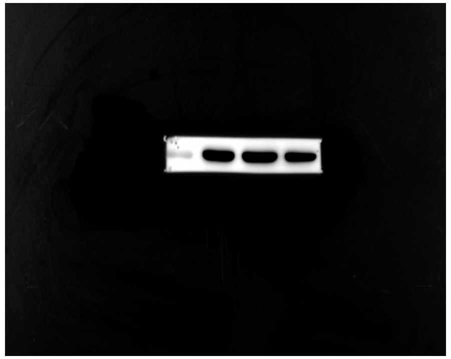

Figure 7A-21g-USP43

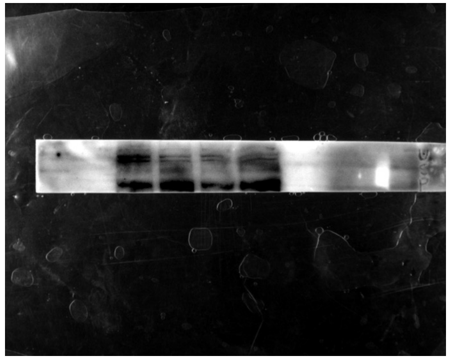

Figure 7A-27-FASN

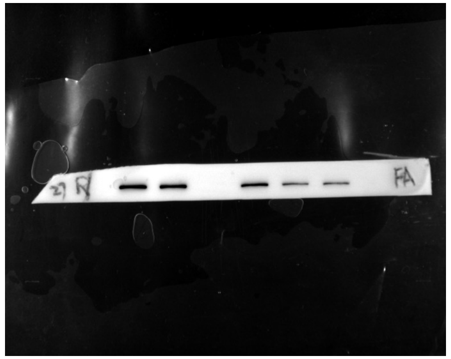

Figure 7A-27-HIF1a

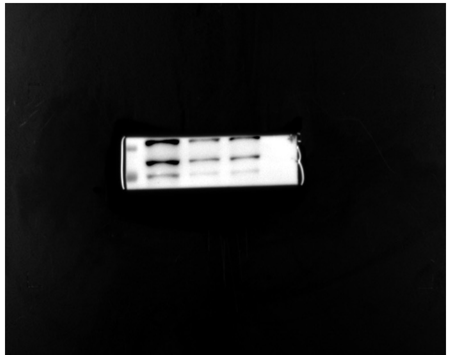

Figure 7A-27-tubulin

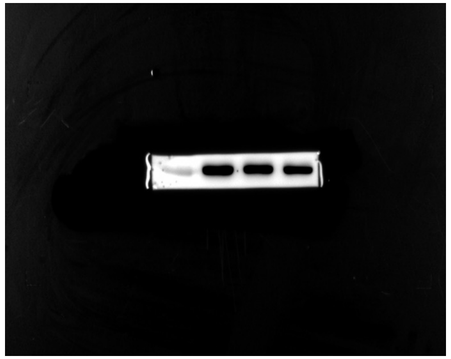

Figure 7A-27-USP43

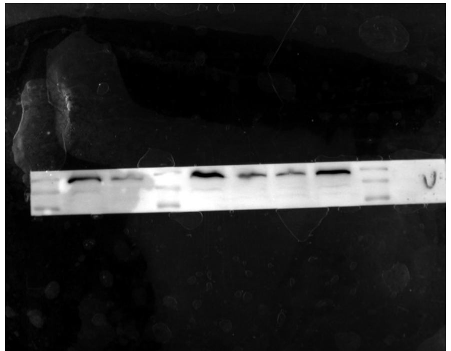

Figure 7B-21g-FASN

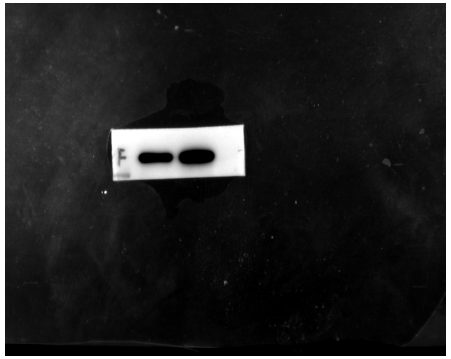

Figure 7B-21g-HIF1a

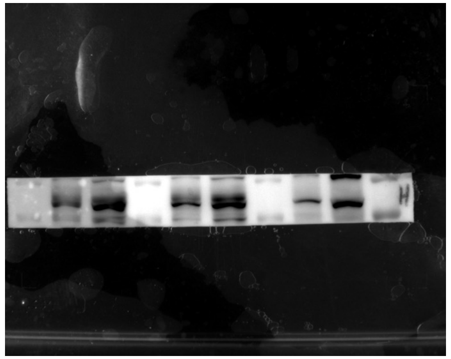

Figure 7B-21g-tubulin

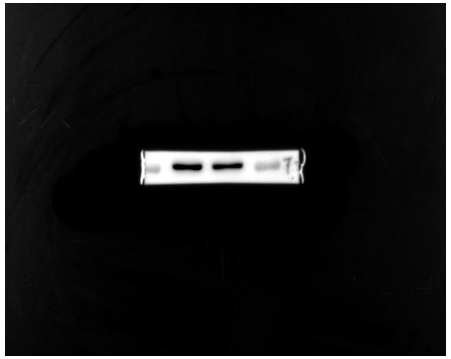

Figure 7B-21g-USP43

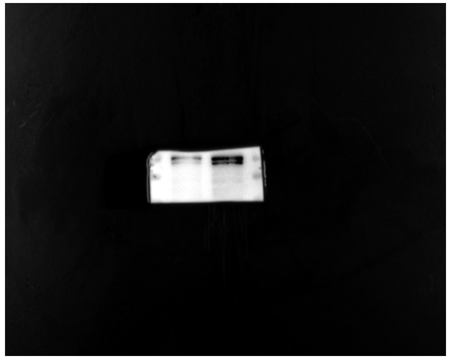

Figure 7B-27-FASN

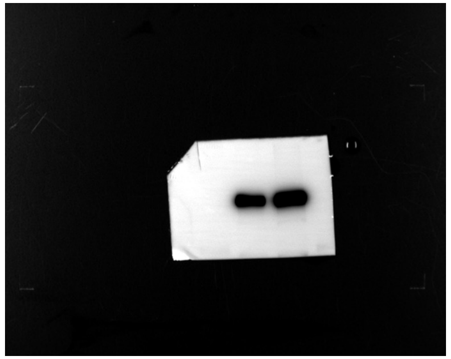

Figure 7B-27-HIF1a

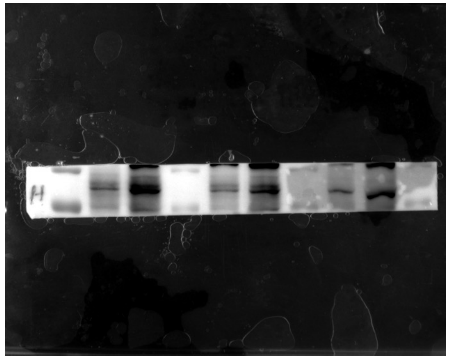

Figure 7B-27-tubulin

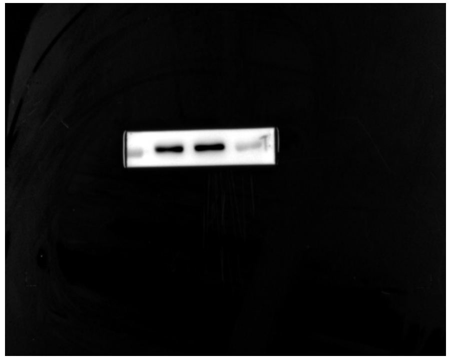

Figure 7B-27-USP43

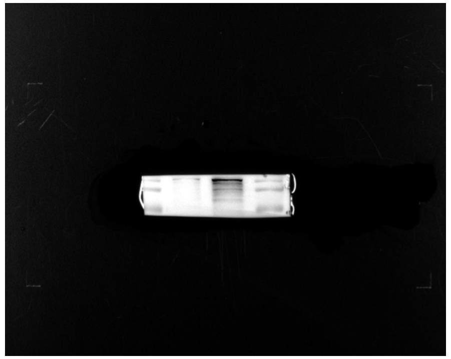

Figure 7C-21g-cytoplasmHIF1a

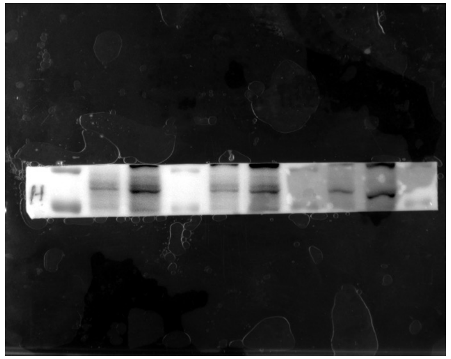

Figure 7C-21g-H3

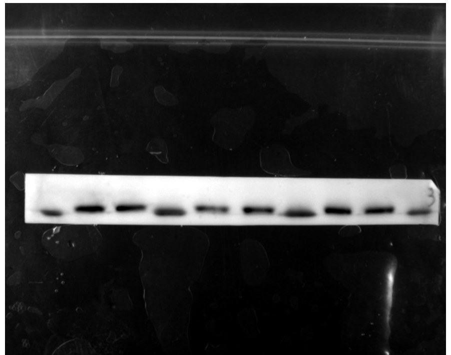

Figure 7C-21g-nucleusHIF1a

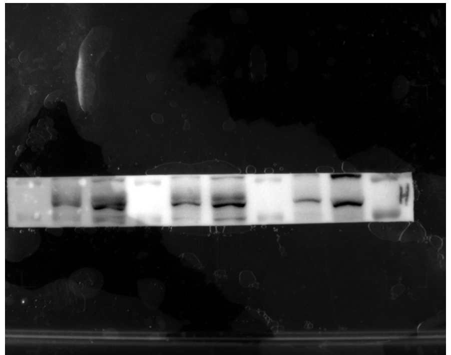

Figure 7C-21g-tubulin

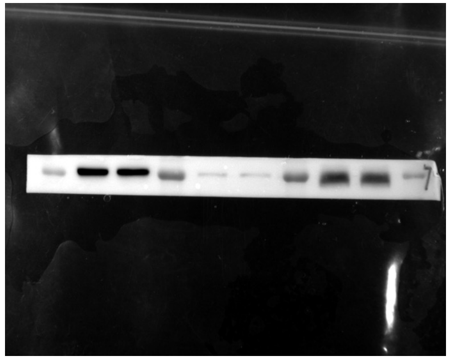

Figure 7C-27-cytoplasmHIF1a

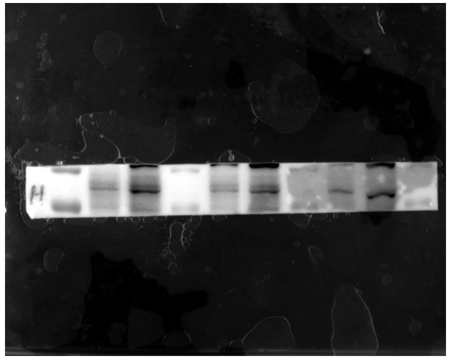

Figure 7C-27-H3

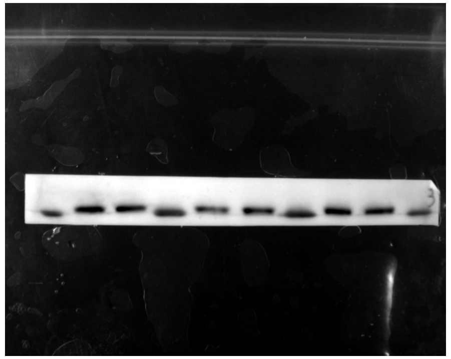

Figure 7C-27-nucleusHIF1a

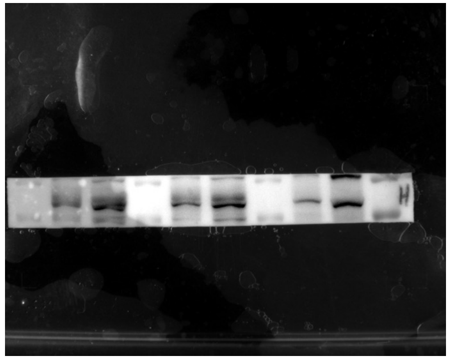

Figure 7C-27-tubulin

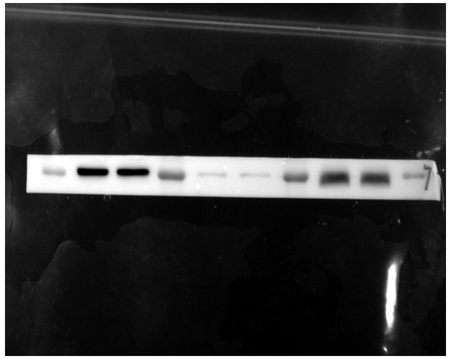

Figure 7E-21g-HIF1a

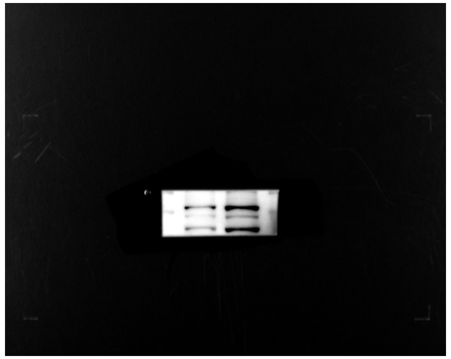

Figure 7E-21g-SLC7A11

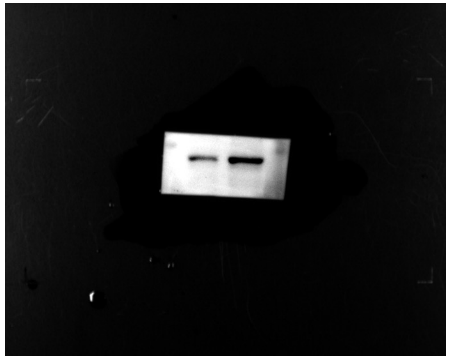

Figure 7E-21g-tubulin

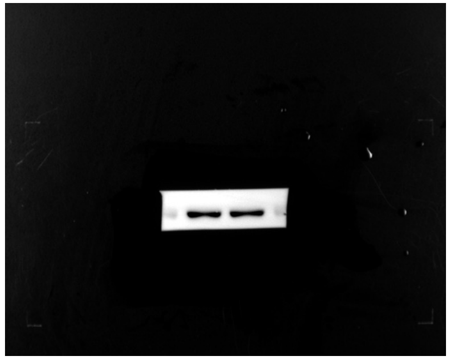

Figure 7E-27-HIF1a

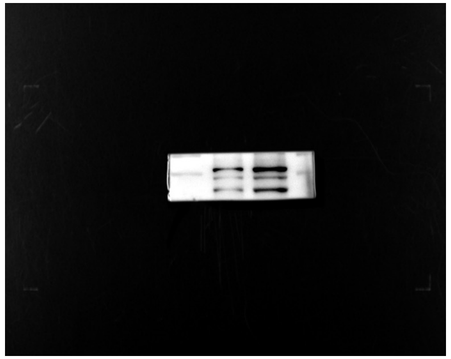

Figure 7E-27-SLC7A11

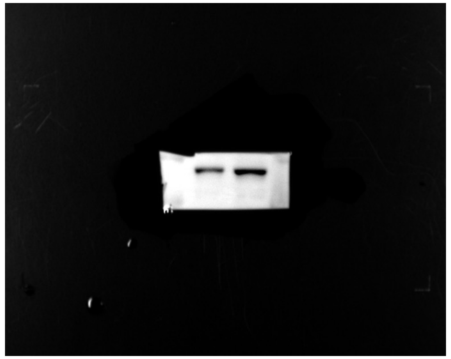

Figure 7E-27-tubulin

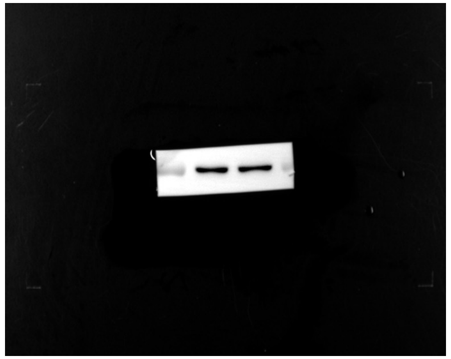

Figure 7H-27-FASN

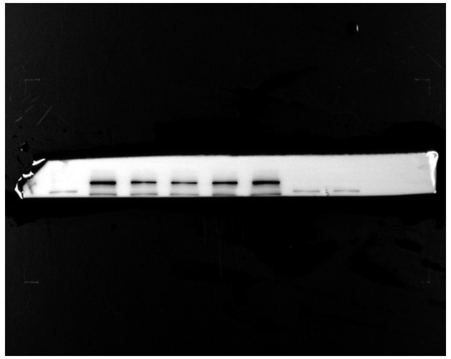

Figure 7H -27-HIF1a

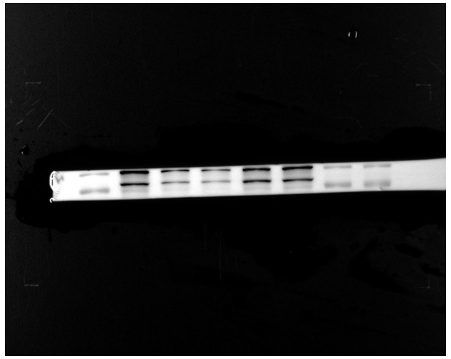

Figure 7H-27-SLC7A11

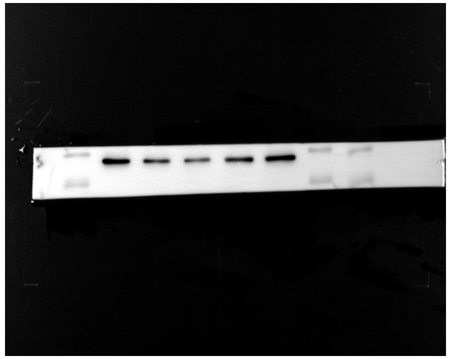

Figure 7H -27-tubulin

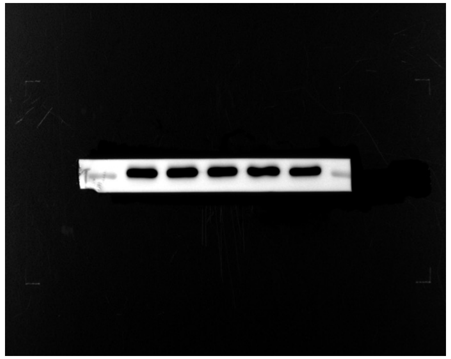

Figure 7H -27-USP43

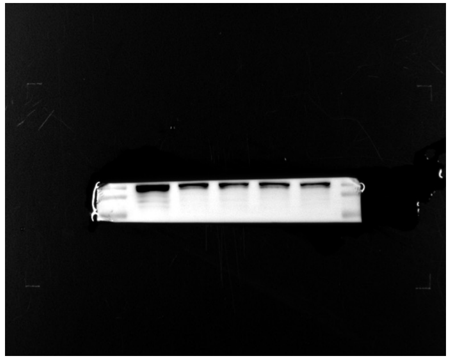

Figure 7I-21g-FASN

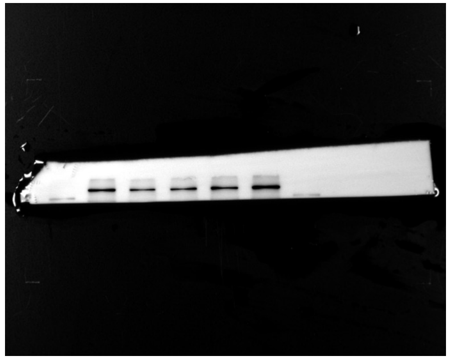

Figure 7I -21g-HIF1a

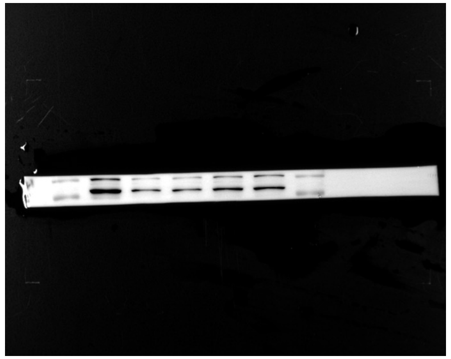

Figure 7I -21g-SLC7A11

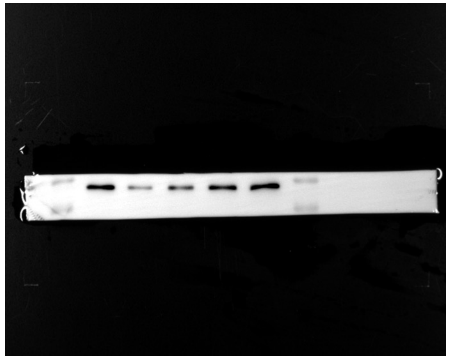

Figure 7I -21g-tubulin

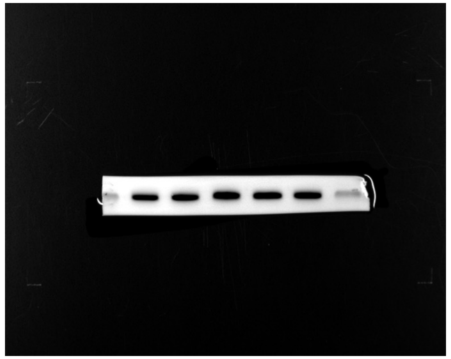

Figure 7I -21g-USP43

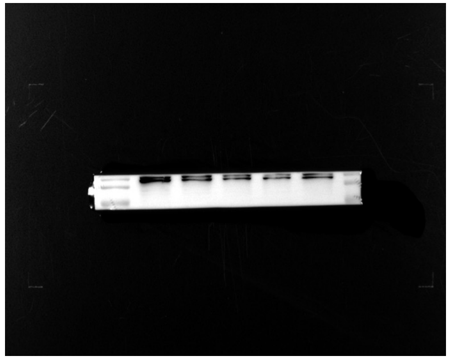

Supplement: Supplementary file 11 — full uncropped Gels and Blots image(s) [file 41419_2025_7886_MOESM11_ESM.pdf]
